# From Primary Tumor to Peritoneal Niche: Microenvironmental Divergence in Gastric Cancer Peritoneal Metastasis

**DOI:** 10.3390/cells15121055

**Published:** 2026-06-09

**Authors:** Catalin-Bogdan Satala, Alina-Mihaela Gurau, Daniela Mihalache, Gabriela Patrichi, Roxana-Cristina Mehedinti, Andy Radu Leibovici, Gabriela Gurău

**Affiliations:** 1 Medical and Pharmaceutical Research Center, Faculty of Medicine and Pharmacy, “Dunărea de Jos” University of Galati, 800008 Galati, Romania; catalin.satala@ugal.ro (C.-B.S.); roxana.mehedinti@ugal.ro (R.-C.M.); gabriela.gurau@ugal.ro (G.G.); 2 Department of Pathology, Clinical County Emergency Hospital Braila, 810325 Braila, Romania; 3 The School for Doctoral Studies in Biomedical Sciences, “Dunărea de Jos” University of Galati, 800008 Galati, Romania; 4 The Doctoral School of Medicine and Pharmacy, “George Emil Palade” University of Medicine, Pharmacy, Science and Technology, 540142 Targu Mures, Romania; gabriela.constantin@umfst.ro; 5 “Sf. Ioan” Clinical Emergency Pediatric Hospital, 800487 Galati, Romania

**Keywords:** gastric cancer, peritoneal metastasis, tumor microenvironment, peritoneal niche, malignant ascites, mesothelial remodeling, cancer-associated fibroblasts, tumor-associated macrophages, immune exclusion, therapeutic resistance

## Abstract

Gastric cancer peritoneal metastasis is not simply an extension of the primary tumor into the abdominal cavity. It represents a biologically distinct disease context shaped by interactions between disseminated tumor cells, peritoneal fluid, mesothelial surfaces, submesothelial stroma, extracellular matrix, immune populations, and malignant ascites. In this narrative review, we examine peritoneal metastasis as a transition between three related but physiologically different states: the primary gastric tumor, free-floating tumor cells or spheroids in the peritoneal fluid, and established mesothelial or submesothelial metastatic implants. We discuss how tumor cells acquire dissemination competence in the primary tumor, survive detachment and fluid-phase stress, adhere to remodeled mesothelium, recruit stromal and immune support, and adapt to ascites-mediated signaling. We also review how the peritoneal niche may contribute to biomarker discordance, immune exclusion, therapeutic resistance, and limitations of conventional response assessment. Where relevant, we distinguish evidence derived directly from gastric cancer peritoneal metastasis from preclinical data, extrapolation from other peritoneal malignancies, and hypothesis-generating interpretation. Finally, we summarize practical implications for tissue sampling, ascites and lavage analysis, biomarker interpretation, translational modeling, and peritoneal-directed therapeutic strategies. A clearer understanding of the biological divergence between the primary tumor, the fluid-phase compartment, and peritoneal implants may improve the study and clinical management of gastric cancer peritoneal metastasis.

## 1. Introduction

Gastric cancer remains one of the leading causes of cancer-related mortality worldwide, largely because many patients are diagnosed with advanced or biologically aggressive diseases. Among the patterns of metastatic spread, peritoneal metastasis is particularly challenging. It is frequently associated with delayed detection, malignant ascites, bowel dysfunction, nutritional decline, limited treatment tolerance, and poor survival. Clinically, the development of peritoneal dissemination often marks a decisive shift in the disease course. Biologically, it reflects more than tumor progression alone: it indicates that gastric cancer cells have entered and adapted to a highly specialized metastatic compartment [1,2,3].

The traditional view of peritoneal dissemination has emphasized tumor cell-intrinsic properties such as invasiveness, epithelial–mesenchymal plasticity, anoikis resistance, and migratory capacity. These features remain important, but they do not fully explain why only some disseminated cells establish clinically relevant peritoneal disease. Tumor cells shed from the primary lesion must survive without stable tissue attachment, persist within the peritoneal cavity, interact with mesothelial surfaces, invade the submesothelial matrix, and acquire stromal and immune support. Each of these steps depends not only on the malignant cell, but also on the biological state of the host compartment in which it arrives [4,5,6].

The peritoneum should therefore not be regarded as a passive surface that receives exfoliated gastric cancer cells. It is a dynamic anatomical and immunobiological niche composed of mesothelial cells, fibroblasts, adipose-associated stromal cells, endothelial structures, extracellular matrix, resident and recruited immune populations, soluble inflammatory mediators, and, in advanced disease, malignant ascites. Under tumor-derived and inflammation-driven pressure, this compartment can be remodeled into a permissive environment that supports adhesion, immune evasion, stromal anchoring, metabolic adaptation, and therapeutic resistance [4,5,7,8].

This perspective has important implications for how gastric cancer peritoneal metastasis is interpreted. Peritoneal disease is often classified clinically as advanced-stage metastasis, but its biology cannot be inferred solely from the primary gastric tumor [9,10,11]. Emerging multi-omic, single-cell, spatial, and immune-profiling studies suggest that peritoneal metastases may differ substantially from the primary lesion in stromal organization, immune contexture, extracellular matrix remodeling, mesothelial interaction, and fluid-phase signaling. These differences may help explain why peritoneal metastasis is frequently associated with poor drug penetration, limited efficacy of systemic therapy, and disappointing responses even in the era of biomarker-driven and immune-based treatment strategies [9,10].

Malignant ascites adds a further layer of complexity. Rather than representing only a late clinical manifestation, ascitic fluid can function as a biologically active compartment. It contains isolated tumor cells, spheroids, immune cells, cytokines, chemokines, extracellular vesicles, metabolites, and stromal mediators that may sustain tumor survival and promote reseeding across peritoneal surfaces. In this setting, peritoneal metastasis is not confined to solid implants. It becomes a surface–fluid disease, maintained through continuous exchange between attached lesions, free-floating tumor aggregates, mesothelial interfaces, and host-derived cellular components [4,12,13,14].

A clearer understanding of the divergence between the primary gastric tumor microenvironment and the peritoneal metastatic niche may therefore improve the interpretation of disease progression, therapeutic resistance, and biomarker performance. Primary tumor profiling remains essential for diagnosis and treatment selection, but it may be insufficient when the dominant clinical problem is peritoneal disease. The peritoneal compartment introduces biological variables that are not fully captured by routine assessment of the primary lesion, including mesothelial remodeling, ascites-mediated signaling, stromal protection, immune exclusion, macrophage-dominated inflammation, and altered drug accessibility [4,10,15].

In this narrative review, we examine gastric cancer peritoneal metastasis as a progressive transition between three related but biologically distinct disease states: the primary gastric tumor, free-floating tumor cells or spheroids in peritoneal fluid, and established mesothelial or submesothelial metastatic implants. This framework is intended to clarify why peritoneal disease cannot be fully interpreted from the primary tumor alone, even when primary-tumor profiling remains essential for diagnosis and treatment selection. Rather than providing another general overview of peritoneal dissemination, this review focuses on the microenvironmental divergence that occurs as gastric cancer cells move from an organ-embedded tumor to a fluid-exposed and surface-based metastatic compartment. We highlight evidence from gastric cancer whenever available, indicate when concepts are extrapolated from other peritoneal malignancies or preclinical models, and discuss the practical implications for sampling, biomarker interpretation, response assessment, translational modeling, and peritoneal-directed therapeutic strategies.

## 2. Literature Search Strategy

This narrative review was prepared through a structured, non-systematic literature search. The purpose of the search was to identify relevant clinical, translational, pathological, and experimental studies addressing gastric cancer peritoneal metastasis and related mechanisms of peritoneal disease.

The literature search was conducted in PubMed, Scopus, Web of Science, and Google Scholar. Articles published in English were considered. Priority was given to studies published within the last ten years, although older landmark publications were included when they provided important clinical, methodological, or mechanistic context. The search was not restricted to a single study design and included original research articles, translational studies, clinical trials, observational studies, preclinical studies, systematic reviews, meta-analyses, and high-quality narrative reviews.

The search strategy used combinations of the following terms: “gastric cancer”, “gastric adenocarcinoma”, “peritoneal metastasis”, “peritoneal dissemination”, “peritoneal carcinomatosis”, “malignant ascites”, “peritoneal lavage”, “positive cytology”, “mesothelial cells”, “mesothelium”, “cancer-associated fibroblasts”, “extracellular matrix”, “tumor-associated macrophages”, “tumor immune microenvironment”, “immune exclusion”, “spheroids”, “anoikis resistance”, “single-cell sequencing”, “spatial transcriptomics”, “biomarkers”, “therapeutic resistance”, “intraperitoneal chemotherapy”, “HIPEC”, and “PIPAC”.

Studies were screened according to their relevance to the scope of the review. Preference was given to articles that directly investigated gastric cancer peritoneal metastasis, paired primary and metastatic samples, peritoneal implants, malignant ascites, peritoneal lavage, cytology, or peritoneal-directed therapeutic approaches. Studies from other peritoneal malignancies were considered only when they addressed mechanisms or methodological issues relevant to peritoneal dissemination and when direct gastric cancer evidence was limited. The selected literature was organized thematically into categories covering clinical behavior and detection of peritoneal metastasis, mechanisms of transcoelomic spread, peritoneal tissue interactions, ascites-based analyses, immune and stromal profiling, biomarker assessment, therapeutic resistance, intraperitoneal treatment strategies, and translational disease models. The literature search aimed to include representative studies published up to March 2026, with emphasis on recent clinical, translational, and experimental evidence relevant to gastric cancer peritoneal metastasis. After removal of duplicates and screening for relevance, 119 articles were assessed in detail. The final selection of references was based on relevance, methodological quality, recency, and contribution to the conceptual aims of the manuscript.

## 3. Peritoneal Metastasis: A Unique Mode of Progression in Gastric Cancer

**Figure 1:**
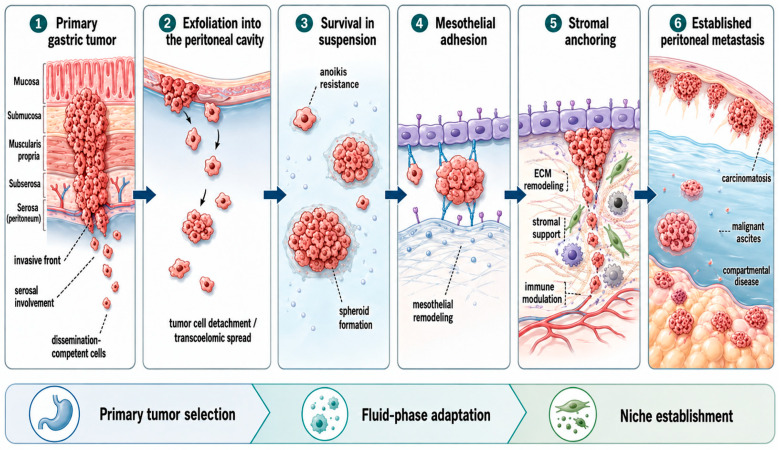
From primary tumor to peritoneal niche. Dissemination-competent tumor cells may emerge at the invasive front and serosa-adjacent regions of the primary tumor, then exfoliate into the peritoneal cavity. After detachment, tumor cells must survive in suspension, resist anoikis, and often form multicellular spheroids. Successful colonization requires adhesion to a remodeled mesothelial surface, access to submesothelial matrix, stromal anchoring, extracellular matrix remodeling, and immune modulation. Established peritoneal metastasis is characterized by surface implants, malignant ascites, floating tumor aggregates, and compartment-wide signaling, reflecting the transition from local tumor escape to a specialized peritoneal metastatic niche.

Peritoneal metastasis occupies a distinct position in the natural history of gastric cancer. Unlike hematogenous dissemination, which requires intravasation, circulation, extravasation, and colonization of distant organs, peritoneal spread develops within a regional but biologically specialized compartment. Tumor cells that reach the peritoneal cavity do not simply relocate to another tissue; they enter a new environment to which they must adapt, defined by mesothelial surfaces, peritoneal fluid dynamics, submesothelial matrix, resident immune populations, adipose-associated structures, and, in many patients, malignant ascites. These features create a set of biological constraints that differ from those encountered in the primary gastric wall or in vascular metastatic sites [3,4,5]. To illustrate this site-specific transition, Figure 1 summarizes the major biological steps through which gastric cancer cells progress from the primary tumor microenvironment to an established peritoneal metastatic niche.

This distinction is clinically relevant because peritoneal metastasis is consistently associated with poor outcomes and limited therapeutic efficacy. Patients frequently develop ascites, bowel dysfunction, impaired oral intake, nutritional decline, and reduced performance status, all of which restrict tolerance to systemic treatment. Contemporary clinical reviews report poor median survival with systemic chemotherapy in gastric cancer peritoneal metastasis, often in the range of only several months, highlighting both the aggressiveness of this phenotype and the limitations of conventional systemic approaches for a compartmentalized disease process [16,17,18].

The poor prognosis of peritoneal dissemination should not be attributed solely to tumor burden. Increasing evidence suggests that peritoneal involvement marks a biologically unfavorable state characterized by site-specific microenvironmental remodeling. Multi-omic analyses of primary gastric tumors, peritoneal metastases, and adjacent peritoneal tissues have shown that peritoneal organotropism is associated with distinct genomic, transcriptomic, and microenvironmental features, supporting the idea that the peritoneal niche is actively shaped during disease progression rather than passively colonized [18]. Immune profiling studies further suggest that peritoneal lesions may exhibit lower CD4-positive and CD8-positive T-cell densities than their paired primary tumors and may be enriched for immune-desert or intrinsically immunosuppressive patterns [19].

### 3.1. Clinical Significance of Peritoneal Dissemination

Peritoneal dissemination is one of the most consequential manifestations of advanced gastric cancer. Its impact is not limited to shortened survival; it also produces a characteristic clinical syndrome. These manifestations often develop in parallel with limited measurable disease on conventional imaging, making peritoneal metastasis both clinically severe and difficult to monitor [3,20].

The burden of peritoneal disease also affects the feasibility of treatment. Patients with symptomatic ascites, bowel dysfunction, or nutritional deterioration may be less able to receive intensive systemic regimens, undergo surgical interventions, or participate in clinical trials. Thus, peritoneal metastasis influences outcome through two mechanisms: intrinsic biological aggressiveness and reduction in therapeutic opportunity. This dual effect partly explains why peritoneal involvement remains a major driver of cancer-related death in gastric cancer despite advances in systemic therapy [3,13,21].

The clinical phenotype of peritoneal metastasis also differs from that of nodal, hepatic, or pulmonary metastases. Peritoneal disease may progress diffusely across serosal surfaces, produce ascites, and impair gastrointestinal function without forming large, easily measurable masses. This diffuse growth pattern can lead to underestimation of disease burden by imaging and delayed recognition of progression. It also complicates response assessment, since changes in ascites, cytology, symptoms, nutritional status, and bowel function may be more clinically meaningful than conventional changes in lesion diameter [3,20].

Recent translational data further support the clinical relevance of the intraperitoneal immune landscape, showing that local immune composition may be associated with treatment response and survival in gastric cancer peritoneal metastasis [22]. These findings reinforce the view that peritoneal metastasis represents a biologically distinct disease context, not only an anatomic category.

### 3.2. Biological Steps of Transcoelomic Spread

Peritoneal dissemination follows a progression route that differs from hematogenous or lymphatic metastasis. Tumor cells destined for the peritoneal cavity must: (i) detach from the primary lesion, (ii) survive loss of anchorage within the peritoneal milieu, (iii) adhere to mesothelial surfaces, (iv) invade through or beneath the mesothelial barrier, and (v) establish stromal support for outgrowth. These steps are sequential but not purely mechanical; at each stage, tumor cell survival depends on interaction with host-derived components of the peritoneal niche [4,5].

Local escape is often associated with deep invasion and serosal involvement, but physical access to the peritoneal cavity is not sufficient. Disseminating cells must acquire properties that support detachment, anoikis resistance, spheroid formation, immune evasion, mesothelial adhesion, and matrix-dependent survival. These transitions are shaped by epithelial–mesenchymal plasticity, altered adhesion, stromal-derived survival signals, tumor-derived cytokines, extracellular vesicles, proteases, inflammatory mediators, fibroblasts, macrophages, endothelial cells, and adipose-associated stromal elements [10,18,23,24,25,26,27,28].

Once metastatic outgrowth is established, malignant ascites can amplify disease by distributing tumor cells, cytokines, extracellular vesicles, metabolites, and immunosuppressive mediators throughout the peritoneal cavity. In this way, transcoelomic dissemination evolves from a local escape event into a compartment-wide disease process [23,26,29].

### 3.3. The Peritoneum as a Permissive Metastatic Soil

The tropism of gastric cancer for the peritoneal cavity cannot be explained by anatomical proximity alone. Although serosal invasion facilitates tumor cell access to the peritoneal space, successful metastasis requires a receptive host environment. The peritoneum becomes permissive through coordinated changes in its mesothelial, stromal, immune, vascular, adipose-associated, and fluid compartments [4,18,26].

The mesothelial interface is central to this process. Rather than functioning as a passive lining, mesothelial cells can respond to tumor-derived signals by losing barrier integrity, increasing adhesive properties, secreting inflammatory mediators, and contributing to matrix remodeling. These changes may allow tumor cells to attach more efficiently and gain access to the submesothelial space. In this sense, the peritoneal surface is not merely a landing site; it is an active regulator of whether tumor cell contact becomes metastatic implantation [4,5,7].

The submesothelial compartment then provides the structural basis for early colonization. Fibroblasts and extracellular matrix components supply anchorage and survival cues, while macrophages and other immune populations can shape the balance between tumor clearance and tolerance. In gastric cancer peritoneal metastasis, the local immune environment often appears skewed toward ineffective or suppressive inflammation rather than productive antitumor immunity. This may reduce immune-mediated elimination of early implants and facilitate stromal remodeling [18,26,27].

Malignant ascites further expands the niche beyond attached lesions. Ascitic fluid can sustain tumor spheroids, distribute soluble mediators, impair immune function, and condition distant peritoneal surfaces. It creates a fluid phase of disease that is absent from the primary tumor and that may contribute to continuous reseeding. The peritoneal ecosystem, therefore, includes both solid and liquid components, each reinforcing the other [4,26,29,30,31,32].

## 4. The Primary Gastric Tumor Microenvironment: The Point of Departure

Any discussion of peritoneal dissemination in gastric cancer must begin with the primary tumor, not because it fully predicts peritoneal disease, but because it provides the first microenvironmental context in which dissemination-competent states may emerge. The primary gastric tumor is not a homogeneous epithelial mass. It is a spatially organized tissue ecosystem in which malignant cells interact with fibroblasts, immune cells, endothelial structures, extracellular matrix, and soluble mediators. These interactions influence invasion, immune recognition, stromal remodeling, treatment sensitivity, and ultimately the probability that tumor cells will acquire the capacity to leave the gastric wall [33,34,35,36,37,38].

This is particularly important for peritoneal dissemination. Tumor cells that eventually reach the peritoneal cavity are unlikely to represent a random sample of the primary lesion. They are more likely to arise from regions where malignant cells are exposed to stromal activation, inflammatory signaling, matrix remodeling, hypoxia, immune pressure, and serosal proximity. These local conditions may favor phenotypes capable of detachment, stress tolerance, immune escape, and interaction with extracellular matrix. In this sense, the primary tumor creates the biological potential for peritoneal spread, even though the peritoneal niche later transforms that potential into established metastatic disease [26,32,39].

Recent single-cell and spatial analyses support this view by showing that gastric cancers are organized into heterogeneous cellular states and microanatomical regions rather than uniform tumor masses. Such studies have identified distinct stromal subsets, immune neighborhoods, lymphoid aggregates, macrophage–fibroblast interaction modules, and spatially restricted programs associated with prognosis and treatment response [40,41,42]. These observations are relevant to peritoneal metastasis because they suggest that dissemination may be influenced by localized microenvironmental conditions that are not captured by routine sampling or bulk profiling of the primary tumor.

### 4.1. Major Cellular and Spatial Features of the Primary Tumor Microenvironment

The primary gastric tumor microenvironment contains multiple cellular compartments whose biological importance depends on their functional integration. Malignant epithelial cells remain the central population, but they do not act in isolation. They produce cytokines, chemokines, extracellular vesicles, matrix-remodeling signals, and metabolic byproducts that influence surrounding host cells. In turn, stromal and immune components regulate tumor cell survival, invasion, immune visibility, and therapeutic response. This bidirectional communication is already active before overt dissemination and may help define which tumor cell populations acquire metastatic potential [34,43,44].

Fibroblasts are among the most influential stromal populations in gastric cancer. Rather than representing a uniform desmoplastic background, they comprise functionally diverse subsets with different matrix-producing, inflammatory, metabolic, and immunoregulatory roles. High-resolution single-cell analyses have identified multiple fibroblast states in gastric cancer, including subsets associated with poor prognosis, macrophage infiltration, T-cell dysfunction, immune exclusion, and reduced predicted benefit from immunotherapy [45]. Other studies integrating single-cell and bulk transcriptomic data have linked specific fibroblast programs, including IGF1-positive populations, with unfavorable prognosis and reduced chemosensitivity [40].

The relevance of these fibroblast states for peritoneal dissemination is not that they are identical to fibroblasts in peritoneal metastases. Rather, they show that the primary tumor may already harbor stromal programs that support invasion, immune restriction, and treatment resistance. Fibroblast-rich regions can remodel extracellular matrix, increase tissue stiffness, provide motility cues, and create physical or biochemical barriers to immune cell penetration. Such regions may function as protected territories in which malignant cells are exposed to reduced immune pressure and enhanced stromal support [36,45,46].

The immune compartment of the primary gastric tumor is equally heterogeneous. Overall lymphocyte density is insufficient to define immune competence. What matters is whether immune cells are spatially positioned to engage malignant cells, whether antigen-presenting cells support local activation, and whether lymphocytes form coordinated structures or remain excluded from tumor nests. Some gastric cancers contain tertiary lymphoid structure-like regions, activated T-cell populations, B-cell niches, dendritic cells, and high endothelial venule-like structures, suggesting organized local immunity. Others show fragmented, excluded, or dysfunctional immune infiltrates [35,47].

This spatial organization has implications for metastatic selection. An immune-inflamed region may exert pressure that favors tumor cells with immune-escape mechanisms. A stromal-rich, immune-excluded region may allow the survival of cells that interact efficiently with the matrix and fibroblast-derived signals. A poorly immunogenic region may permit expansion with limited immune constraint. Thus, the primary tumor can contain multiple immunological landscapes, each selecting different tumor cell states [36].

Myeloid cells add another layer of microenvironmental complexity. Tumor-associated macrophages and related populations can link inflammation, fibroblast activation, angiogenesis, matrix remodeling, and immunosuppression. In gastric cancer, spatially organized macrophage–fibroblast circuits have been associated with aggressive behavior and poor outcome, including interactions between SPP1-positive macrophage programs and collagen-related stromal pathways. These findings suggest that the primary tumor may already contain multicellular modules capable of coordinating invasion and immune dysfunction [36,48].

Endothelial and vascular-associated populations should also be considered part of this organization. Beyond providing perfusion, vascular structures regulate immune cell trafficking, hypoxia, stromal activation, and therapeutic access. Spatial analyses increasingly suggest that immune organization and stromal density are linked to local vascular architecture. Therefore, the vascular compartment contributes not only to nutrient delivery, but also to the territorial organization of the primary tumor microenvironment [34,38].

Extracellular matrix integrates many of these interactions. Its composition, alignment, stiffness, and proteolytic remodeling influence tumor cell migration, immune infiltration, oxygen diffusion, and drug distribution. Fibroblast-derived matrix programs, together with macrophage-associated remodeling, may generate microregions that favor invasion and immune protection, particularly at invasive fronts and serosa-adjacent regions [49,50]. Together, these findings support the view that peritoneal dissemination may emerge from specific microenvironmental contexts within the primary lesion, rather than from the tumor mass as an average.

### 4.2. Immune–Stromal Organization and Metastatic Competence

The ability of gastric cancer cells to disseminate to the peritoneum is shaped by the local conditions under which they evolve within the primary tumor. Metastatic competence is unlikely to emerge from a neutral background. It is more plausibly selected in regions where malignant cells are exposed to stromal remodeling, immune pressure, hypoxia, mechanical stress, and altered matrix interactions. These pressures can favor tumor cell states capable of invasion, detachment, survival outside normal tissue architecture, and cooperation with host-derived compartments [5,23].

The invasive front is particularly important. It represents the interface between malignant tissue and host structures, where tumor cells interact directly with activated fibroblasts, macrophages, endothelial cells, remodeled matrix, and inflammatory mediators [39]. In this region, tumor cells may acquire partial epithelial–mesenchymal plasticity without fully losing epithelial identity. Such intermediate states may be especially relevant for peritoneal dissemination because they combine motility, adhesive flexibility, stress tolerance, and the ability to re-establish growth after implantation [44].

Serosa-adjacent regions may be even more relevant to peritoneal spread. Tumor cells approaching the serosal surface are exposed to stromal and inflammatory programs that may partially resemble the conditions they will later encounter in the peritoneal cavity: altered matrix contacts, local tissue disruption, inflammatory mediators, and proximity to mesothelial structures. Although the peritoneal niche ultimately imposes distinct pressures, the primary tumor may preselect cells capable of responding to surface-based and matrix-dependent survival cues [4,5].

Immune organization contributes to this selection. A primary tumor with coordinated antigen presentation and effective cytotoxic infiltration may restrain dissemination or select for immune-resistant subclones. Conversely, a tumor dominated by immune exclusion, T-cell dysfunction, suppressive macrophages, or regulatory populations may allow invasive tumor cells to survive in protected regions. Importantly, immune suppression in the primary tumor is rarely driven by a single mechanism. It often emerges from the combined effects of stromal barriers, myeloid populations, inhibitory cytokines, dysfunctional antigen presentation, and metabolic constraints [51,52].

Stromal organization reinforces this immune effect. Fibroblast-rich regions can generate extracellular matrix tracks, provide motility cues, and restrict lymphocyte access. Dense or aligned matrix can separate effector cells from tumor nests, while fibroblast-derived chemokines may recruit suppressive myeloid cells or alter T-cell localization. As a result, stromal remodeling and immune dysfunction become mutually reinforcing. A region that supports invasion mechanically may simultaneously protect tumor cells immunologically [36,47].

These local immune–stromal configurations may help explain why tumors with similar anatomical stage can follow different metastatic trajectories. Conventional pathological features such as depth of invasion, serosal involvement, histological subtype, and lymphovascular invasion remain important, but they may not fully capture the microenvironmental conditions that permit peritoneal dissemination. Two tumors with comparable stage may differ substantially in stromal architecture, immune accessibility, macrophage–fibroblast interactions, and matrix organization [32,48].

This point is relevant for biomarker interpretation. Bulk profiling of the primary tumor may identify inflammatory or stromal signatures, but it often loses spatial information. It may not reveal whether cytotoxic T cells are in contact with tumor cells, whether macrophages are concentrated at invasive margins, whether fibroblast subsets form barriers around epithelial nests, or whether matrix remodeling is localized near serosal invasion. For peritoneal metastasis, such spatial details may be critical because the cells that disseminate may arise from restricted niches within the primary tumor [32,36].

The primary tumor may also condition the peritoneal compartment before macroscopic metastasis becomes evident. Tumor-derived cytokines, chemokines, extracellular vesicles, and other soluble mediators can act beyond the primary lesion and may contribute to mesothelial activation, inflammatory recruitment, or early niche conditioning. This concept remains difficult to prove in patients, but it provides a plausible link between local tumor biology and later peritoneal receptivity. Peritoneal dissemination may therefore begin biologically before it is visible anatomically [4,53].

### 4.3. Biological Divergence Between the Primary Lesion and Peritoneal Disease

The primary gastric tumor is often treated as the biological reference point for the entire disease course. It is usually the most accessible tissue; it defines histology and molecular subtypes, and it provides material for many therapeutic decisions. In the context of peritoneal dissemination, however, this convention has clear limitations. The primary tumor remains informative, but it is not biologically sufficient [4,32].

Peritoneal metastasis does not simply represent the primary tumor displaced onto another surface. It reflects a transition from an organ-embedded tumor to a compartmental disease state shaped by mesothelial interfaces, fluid-phase tumor survival, ascitic mediators, stromal reorganization, and local immune failure. The biology that determines peritoneal progression is therefore only partly inherited from the primary lesion. It is also produced by the metastatic site [4,26,32].

This discrepancy begins with selection. The cells that reach and colonize the peritoneum are unlikely to represent the average tumor cell population within the gastric wall. They are selected through local invasion, serosal proximity, detachment, survival in suspension, immune exposure, and interaction with peritoneal surfaces. A biopsy from the primary tumor may capture molecular identity, but not necessarily the specific tumor cell states or microenvironmental regions that give rise to peritoneal disease [4].

The immune compartment illustrates this problem. An immune-inflamed primary tumor does not necessarily imply an immune-inflamed peritoneal metastasis. The primary lesion may contain lymphoid aggregates, activated T cells, or antigen-presenting niches, while peritoneal deposits may be lymphocyte-poor, stromal-shielded, or dominated by suppressive myeloid populations. For immunotherapy, the decisive question is not only whether the tumor is antigenic, but whether effector immune cells can reach and function within the lesions driving progression. In patients with peritoneal metastasis, that site is often the peritoneal compartment, not the primary gastric wall [8,32].

The same applies to stromal biology. Fibroblasts and extracellular matrix in the gastric wall are embedded within organ architecture. In the peritoneum, stromal support is assembled around mesothelial surfaces, submesothelial matrix, omental and adipose-associated structures, and ascitic mediators. Even when both sites appear stromal-rich, their functional meanings differ. In the primary tumor, stroma may support invasion; in the peritoneum, it may determine implantation, immune exclusion, drug penetration, and maintenance of microscopic disease [4,32].

This creates a central interpretive problem for translational studies. Primary tumor signatures may predict the risk of peritoneal recurrence, but they may not identify the mechanisms required to maintain established peritoneal metastasis. Risk biology and niche biology overlap, but they are not identical. A primary tumor feature may indicate that dissemination is likely; a peritoneal feature may explain why disseminated cells survive and resist therapy [4,32].

The distinction is particularly important in biomarker-driven treatment. HER2, PD-L1, mismatch repair status, CLDN18.2, FGFR2b, MET, and other markers are often assessed in primary tissue. Yet the response of peritoneal metastases may be limited by stromal density, poor vascularization, immune exclusion, spheroid architecture, ascites-mediated survival signals, or inadequate drug distribution. Resistance may therefore arise not only from tumor cell genotype, but also from the metastatic environment [19,54].

For this reason, peritoneal disease should be studied directly whenever feasible. Peritoneal biopsies, ascites, lavage fluid, cytology cell blocks, single-cell profiling, and spatial analyses provide access to the disease state that actually determines peritoneal progression. Ascites is especially important because it captures a fluid microenvironment with no true equivalent in the primary tumor. Ignoring this compartment risks reducing peritoneal metastasis to attached lesions alone, when its biology is distributed across both solid and liquid phases [32,55].

## 5. The Peritoneal Metastatic Microenvironment

**Figure 2:**
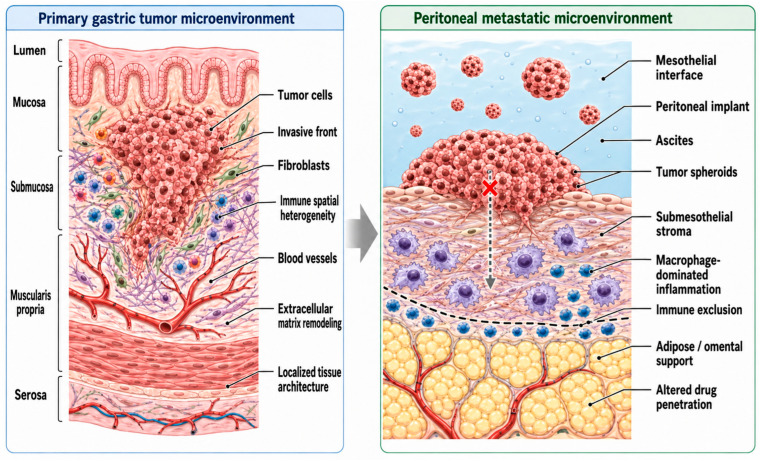
Primary gastric tumor microenvironment versus peritoneal metastatic microenvironment. The primary gastric tumor and the peritoneal metastatic niche differ not only in cellular composition, but also in spatial organization and biological function. Whereas the primary tumor evolves within the structured layers of the gastric wall, peritoneal metastasis develops on mesothelial surfaces and within ascitic fluid. This shift changes how tumor cells interact with stroma, immune cells, extracellular matrix, adipose-associated structures, and therapeutic agents, creating a surface–fluid metastatic niche that is biologically distinct from the organ-embedded primary lesion.

Once gastric cancer cells enter the peritoneal cavity, they encounter a compartment that differs fundamentally from the gastric wall. The peritoneal metastatic niche is organized around surfaces, fluid movement, mesothelial barriers, submesothelial matrix, resident immune cells, adipose-associated structures, and, in advanced disease, malignant ascites. These features create a biological setting in which tumor cells must survive outside their original tissue architecture before they can establish a new one [4,32]. The major differences between the primary gastric tumor microenvironment and the peritoneal metastatic niche are summarized in Figure 2.

The peritoneal microenvironment also changes the scale of disease. In the primary tumor, interactions are largely organized within a tissue mass. In peritoneal metastasis, tumor cells may exist simultaneously as attached implants, microscopic deposits, free-floating cells, and multicellular spheroids. Ascitic fluid can connect anatomically distant surfaces and distribute tumor-derived and host-derived signals throughout the cavity. As a result, peritoneal disease may behave as a compartmental process even when individual lesions appear small or radiologically subtle [29,32].

The following sections examine the major biological components that make this compartment permissive: the mesothelial interface, stromal and extracellular matrix remodeling, myeloid and lymphoid dysfunction, malignant ascites, and metabolic adaptation. Together, these elements explain why peritoneal metastasis cannot be understood as a direct copy of the primary gastric tumor and why it often becomes resistant to therapies selected on the basis of primary tumor features alone [4,29,32].

### 5.1. The Mesothelial Interface: From Protective Barrier to Metastatic Substrate

The mesothelium is the first organized cellular barrier encountered by gastric cancer cells entering the peritoneal cavity. Its role in peritoneal metastasis is therefore more than anatomical. Under physiological conditions, mesothelial cells form a lubricated, anti-adhesive, immunologically responsive surface that limits friction, regulates fluid exchange, and helps preserve peritoneal homeostasis. This surface is not naturally designed to support tumor attachment. For disseminated gastric cancer cells to implant, the mesothelial interface must lose part of its protective function and acquire features that favor adhesion, invasion, and local remodeling [4,7].

This transition is a decisive early event. Tumor cells may exfoliate from a serosa-invasive primary lesion and enter the peritoneal cavity, but their presence in the cavity does not guarantee metastasis. A free-floating cell or spheroid must attach to a surface that normally resists stable cellular adhesion. The conversion of the mesothelium from a protective boundary into a receptive substrate is therefore one of the first steps that separates transient dissemination from true colonization [7,26].

**Figure 3:**
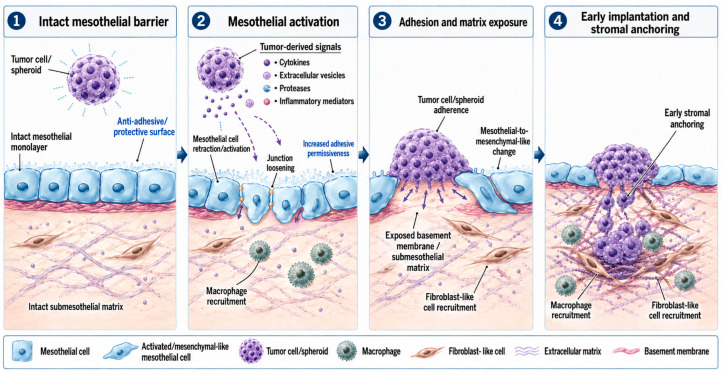
Mesothelial remodeling during early peritoneal implantation. An intact mesothelial monolayer normally acts as a protective, anti-adhesive barrier. Tumor-derived signals, extracellular vesicles, proteases, and inflammatory mediators can induce mesothelial activation, junctional loosening, cell retraction, and increased adhesive permissiveness. These changes expose the basement membrane and submesothelial matrix, enabling tumor cell adhesion, mesothelial-to-mesenchymal-like changes, recruitment of macrophages and fibroblast-like cells, extracellular matrix remodeling, and early stromal anchoring.

Mesothelial remodeling can be induced by multiple tumor-derived and inflammation-associated signals. Cytokines, chemokines, extracellular vesicles, proteases, oxidative stress, and mechanical irritation may alter mesothelial cell phenotype. Activated mesothelial cells may retract, weaken intercellular junctions, expose basement membrane or submesothelial matrix, upregulate adhesion molecules, and secrete inflammatory mediators. These changes allow tumor cells to establish contact with matrix components and gain access to survival signals that are unavailable in suspension [4,7,26]. The sequential changes that convert the mesothelial surface from a protective barrier into a permissive substrate for tumor implantation are illustrated in Figure 3.

The biological significance of this process is that the mesothelial barrier is not only breached but also functionally reprogrammed. A mechanically damaged surface might permit occasional attachment, but sustained metastatic growth requires a more coordinated change in the local environment. Activated mesothelial cells can contribute to extracellular matrix deposition, recruit inflammatory and stromal cells, and promote a wound-healing-like response. Tumor cells exploit this repair program, converting a response intended to restore tissue integrity into one that supports implantation [7,56].

Mesothelial-to-mesenchymal transition-like changes may contribute to this process. Under tumor-derived or inflammatory stimulation, mesothelial cells can acquire features associated with motility, contractility, matrix production, and fibroblast-like behavior. This phenotypic shift can weaken the integrity of the mesothelial monolayer while expanding the stromal support available to early metastatic deposits. The mesothelium, therefore, becomes more than a damaged barrier; it may become a source of tumor-supportive stromal activity [5].

Tumor–mesothelial adhesion is mediated by several classes of interactions, including integrin–matrix binding, CD44–hyaluronan interactions, selectin-related adhesion, cadherin changes, and tumor-induced exposure of submesothelial matrix. However, these molecular events should not be interpreted as isolated ligand–receptor interactions. Their relevance depends on the broader tissue context. Adhesion becomes biologically meaningful when mesothelial activation, matrix exposure, inflammatory recruitment, and tumor cell survival programs converge at the same site [7,27].

The peritoneal surface is also regionally heterogeneous. Tumor implantation is not evenly distributed throughout the cavity. Fluid flow, gravity, lymphatic drainage, mechanical contact, local inflammation, adipose-associated structures, and omental immune aggregates may all influence where tumor cells are retained and where mesothelial remodeling becomes most permissive. Thus, the mesothelium should not be imagined as a uniform sheet. It is a regionally variable interface whose susceptibility to tumor colonization depends on local anatomy and host response [4,7,27].

Once adhesion occurs, the next challenge is invasion into or beneath the mesothelial layer. Tumor cells can produce proteases and matrix-remodeling factors, but host cells also contribute to this process. Activated mesothelial cells, fibroblasts, macrophages, and neutrophil-like inflammatory cells may generate signals that promote matrix degradation and submesothelial access. Peritoneal invasion is therefore a multicellular event. The tumor cell initiates colonization, but the host surface provides part of the machinery that enables it [7,8].

The submesothelial compartment then becomes the early metastatic bed. It contains extracellular matrix, fibroblasts, vascular and lymphatic elements, immune cells, and, depending on the anatomical site, adipose-associated stromal structures. For a tumor cell, entry into this compartment marks a shift from unstable fluid-phase survival to tissue-anchored persistence. Mesothelial remodeling, therefore, functions as a gatekeeping event: it determines whether disseminated tumor cells remain temporary occupants of the peritoneal cavity or become integrated into a supportive stromal niche [4,26].

The immunological role of the mesothelium is also important. Mesothelial cells can regulate leukocyte adhesion, migration, cytokine production, and inflammatory tone. In cancer, this immunoregulatory function may be redirected. Rather than supporting effective antitumor immunity, activated mesothelial cells may participate in the recruitment of myeloid cells, the maintenance of chronic inflammation, and the formation of a local environment that favors tolerance and repair. This provides a link between surface remodeling and immune escape at the earliest stages of implantation [7,26,57,58,59,60].

### 5.2. Stromal Remodeling and Extracellular Matrix Architecture in Peritoneal Metastasis

If mesothelial remodeling allows gastric cancer cells to attach to the peritoneal surface, stromal remodeling determines whether that attachment becomes a durable metastatic lesion. Early contact with activated mesothelium or exposed matrix may provide the first anchoring signals, but sustained growth requires construction of a supportive tissue environment. Peritoneal metastasis is therefore not only a problem of tumor cell adhesion; it is a problem of stromal acquisition [4,30,61].

The first requirement is stable matrix engagement. Once the mesothelial barrier is disrupted or activated, components of the basement membrane and submesothelial matrix become accessible. Tumor cells can then use integrins and other matrix-binding programs to shift from fluid-phase survival to tissue anchorage. This transition is biologically important because extracellular matrix engagement activates survival pathways, reorganizes the cytoskeleton, modifies mechanotransduction, and can reduce vulnerability to apoptosis and therapy-induced stress [7,18,62].

Cancer-associated fibroblast-like populations are central to this transition, although their origin in peritoneal metastasis may be diverse, including resident submesothelial fibroblasts, mesothelium-derived or mesothelium-conditioned cells, adipose-associated stromal cells, recruited precursors, and tumor-conditioned local stromal cells. Their shared relevance lies in matrix production, trophic signaling, immune modulation, and stabilization of early implants [4,7,18].

Extracellular matrix is the structural expression of this stromal adaptation. Collagen deposition, fibronectin accumulation, matrix crosslinking, fiber alignment, and proteolytic remodeling all influence how tumor cells survive and expand. In peritoneal disease, matrix remodeling has a particularly strategic role: it converts a flat serosal surface into a three-dimensional niche capable of sustaining tumor growth. The matrix is not simply a scaffold. It regulates integrin signaling, growth factor availability, tissue stiffness, oxygen diffusion, immune cell movement, and drug penetration [18,29].

The matrix-rich character of peritoneal implants also affects immune surveillance. Dense or aligned extracellular matrix can keep lymphocytes at the periphery of lesions, limit contact between effector cells and tumor cells, and favor accumulation of macrophages or other repair-oriented immune populations. In this way, stromal architecture converts inflammation into a poorly cytotoxic response. The result is not necessarily absence of immune cells, but spatial immune inefficiency. Tumor cells may be surrounded by a biologically active microenvironment that nevertheless fails to eliminate them [30,63].

Fibroblast–macrophage communication is likely to be a major organizer of this state. Fibroblasts can recruit and retain myeloid cells through chemokines and matrix-associated signals, while macrophages can sustain fibroblast activation through pro-fibrotic, angiogenic, and inflammatory mediators. Together, these populations can generate a wound-healing-like environment that supports implantation, suppresses effective lymphocyte activity, and reinforces matrix deposition. In peritoneal metastasis, this cooperation is especially relevant because the metastatic lesion must build and maintain its own stromal foundation [42,61,64].

The omentum and other adipose-rich peritoneal sites add a further dimension. These regions contain adipocytes, stromal progenitors, immune aggregates, vascular structures, and extracellular matrix, making them favorable environments for metastatic implantation. Adipose-associated stromal cells may differentiate into fibroblast-like populations, provide trophic factors, and participate in matrix remodeling. At the same time, adipocytes can supply metabolic substrates that support tumor survival. Thus, in omental disease, stromal remodeling and metabolic support are closely linked [18,65,66].

Angiogenesis is also required for progression beyond microscopic deposits. Early peritoneal implants may initially survive through diffusion and local stromal support, but expanding lesions need vascular adaptation. Tumor cells, fibroblasts, macrophages, mesothelial-derived cells, and adipose-associated populations can all contribute pro-angiogenic signals. The resulting vasculature is often abnormal and heterogeneous, promoting hypoxia, vascular permeability, ascites formation, and uneven drug delivery. Thus, vascular remodeling supports growth while also contributing to the clinical and therapeutic complexity of peritoneal disease [4,18,65,66].

The stromal architecture of peritoneal metastasis has direct pharmacological consequences. Systemic therapies must reach surface-based deposits that may be poorly vascularized, embedded in dense matrix, or protected within fibrotic niches. Even intraperitoneal therapies, which increase local exposure, must penetrate tumor nodules, matrix layers, and spheroid-like structures. Drug concentration in the peritoneal cavity does not guarantee drug access to every viable tumor cell. The physical and biochemical properties of the stroma therefore influence treatment response independently of tumor genotype [4,58,63,67].

This is one reason why biomarker interpretation based only on epithelial targets may be incomplete. A peritoneal lesion may express a therapeutic target, but stromal density, poor perfusion, matrix barriers, and survival signals from fibroblasts or macrophages may limit effective killing. Conversely, targeting the stroma without understanding its functional state may be problematic, because some stromal responses may initially restrain tumor spread or reflect host repair. The goal is unlikely to be the elimination of stromal cells. A more plausible therapeutic strategy is stromal modulation: reducing excessive matrix protection, improving immune access, normalizing vascular function, or disrupting tumor–matrix survival pathways [18,63,68,69].

The temporal evolution of stromal remodeling should also inform treatment. Early peritoneal disease may be most vulnerable to interventions that block adhesion, matrix engagement, or fibroblast recruitment. Established carcinomatosis may require strategies that modify mature collagen-rich stroma, suppress fibroblast–macrophage circuits, improve drug penetration, or reverse immune exclusion. Minimal residual disease may persist in small stromal niches that are not radiologically visible but are sufficient to cause recurrence. Recognizing these stage-dependent stromal states may help explain why therapies effective in low-volume disease often perform less well in advanced carcinomatosis [61,63].

### 5.3. Myeloid Reprogramming and Macrophage-Dominated Immune Suppression

The peritoneal metastatic niche is not immunologically inert, but its inflammatory activity often fails to produce effective tumor rejection. One explanation is the dominance of myeloid programs that convert inflammation into repair, remodeling, and immune restraint. Resident and recruited macrophages can respond to tumor-derived cytokines, chemokines, extracellular vesicles, metabolites, and damage-associated signals, shifting the peritoneal compartment toward functions that support metastatic persistence rather than tumor clearance [4,9,26,70].

The classical M1/M2 framework is insufficient to describe this process. Macrophages in peritoneal metastasis may simultaneously express inflammatory mediators, matrix-remodeling enzymes, angiogenic factors, scavenger receptors, inhibitory ligands, and chemokines that regulate lymphocyte and fibroblast behavior. Their phenotype may differ according to location: suspended in ascites, associated with tumor spheroids, positioned at the mesothelial surface, embedded in stromal implants, or localized within omental adipose tissue. It is therefore more useful to consider macrophages as plastic regulators of the niche rather than as fixed polarization states. Although macrophages represent a dominant myeloid component in many descriptions of gastric cancer peritoneal metastasis, neutrophils may also contribute to peritoneal niche formation. Neutrophil-derived cytokines, chemokines, proteases, reactive oxygen species, and angiogenic mediators can promote inflammation, extracellular matrix remodeling, vascular activation, and tumor cell survival. In the peritoneal cavity, neutrophils may also interact with ascitic fluid components and tumor spheroids, potentially supporting immune suppression, implantation, and early metastatic outgrowth. However, direct evidence defining neutrophil functions specifically in gastric cancer peritoneal metastasis remains more limited than for macrophage-centered mechanisms. Therefore, neutrophils should be considered an important but still incompletely characterized component of the peritoneal inflammatory niche [4,71,72].

In early dissemination, macrophages may influence whether free gastric cancer cells are cleared or supported. In principle, they can phagocytose abnormal cells, present antigen, and initiate adaptive immune responses. However, tumor cells that resist elimination can exploit macrophage-derived survival factors, proteases, and inflammatory mediators. Macrophages may cluster around tumor aggregates, promote spheroid viability, and facilitate mesothelial disruption or matrix exposure. Thus, the same cells that participate in surveillance can become early collaborators in implantation [4,71].

A major consequence of myeloid reprogramming is defective adaptive immunity. Tumor-associated macrophages can suppress T-cell function through inhibitory cytokines, checkpoint ligand expression, nutrient competition, arginine metabolism, prostaglandin production, and recruitment of regulatory populations. They can also interfere with antigen presentation by maintaining tolerogenic or poorly costimulatory conditions. In peritoneal metastasis, these effects are amplified by the fact that macrophages operate across both solid and fluid compartments. A macrophage-conditioned ascitic milieu can impair lymphocytes even before they reach tumor deposits [26,29,73,74].

This distinction helps explain why inflammation in peritoneal disease may be clinically misleading. The peritoneal cavity can appear inflamed, but the dominant immune program may resemble chronic wound repair rather than cytotoxic tumor rejection. Macrophages contribute to this state by promoting vascular permeability, extracellular matrix remodeling, ascites formation, and suppression of effector lymphocyte activity. The result is an immune environment that is biologically active but poorly protective [73,75,76].

Ascites is a major amplifier of myeloid reprogramming. Tumor-derived cytokines, lactate, lipids, extracellular vesicles, hypoxia-associated factors, and cellular debris can condition macrophages toward tumor-supportive states. In turn, macrophages release mediators into the fluid that support spheroid survival, mesothelial activation, fibroblast recruitment, angiogenesis, and T-cell dysfunction. Because ascites circulates throughout the peritoneal cavity, this feedback loop can extend beyond individual implants and influence the compartment as a whole [63,76].

Omental and adipose-rich sites provide an additional context for macrophage activity. In these regions, macrophages are exposed to lipids, adipokines, stromal progenitors, and immune aggregates. Tumor cells, adipocytes, and macrophages can form metabolic-inflammatory circuits that favor implantation and growth. Lipid-loaded or metabolically adapted macrophages may acquire functions that support tumor survival and suppress antitumor immunity, linking the metabolic features of the peritoneal niche to immune failure [76,77,78].

Macrophages also contribute to angiogenesis and ascites formation. By producing VEGF and other pro-angiogenic mediators, they support vascular development within metastatic deposits. However, the resulting vasculature is often abnormal, leaky, and spatially heterogeneous. This can worsen hypoxia, increase vascular permeability, promote fluid accumulation, and contribute to uneven drug delivery. Thus, macrophage-driven angiogenesis supports growth while reinforcing several features that make peritoneal disease difficult to treat [15,75,76].

The role of macrophages in tumor spheroid biology is increasingly relevant. Spheroids in ascites can resist anoikis and therapy through cell–cell adhesion, hypoxia gradients, and partial immune shielding. Macrophages may associate with these aggregates or condition them through soluble mediators, enhancing viability and stress resistance. This suggests that myeloid support is not limited to attached lesions. It may also sustain the mobile, reseeding-competent phase of peritoneal disease [4,19,79,80,81,82].

Macrophage-related biomarkers may help stratify peritoneal disease. The balance between macrophages and effector lymphocytes in ascites, expression of myeloid suppressive programs, cytokine profiles, macrophage localization at tumor–stroma interfaces, and association with matrix-rich regions may all provide information about therapeutic vulnerability. Such biomarkers may be more informative when derived from ascites or peritoneal implants than from the primary tumor alone, because macrophage function is strongly shaped by the local compartment [15,81].

### 5.4. Lymphoid Dysfunction and Immune Exclusion in the Peritoneal Compartment

The lymphoid compartment of gastric cancer peritoneal metastasis illustrates an important principle: the presence of immune cells is not equivalent to effective antitumor immunity. Lymphocytes may be detected in ascites, peritoneal implants, omental tissue, or stromal regions, yet their distribution and functional state often fail to support durable tumor control. Compared with some primary gastric tumors, peritoneal metastases may be less favorable for coordinated adaptive immunity, with reduced lymphocyte access, impaired antigen presentation, stromal exclusion, myeloid suppression, and ascites-associated metabolic stress limiting effective cytotoxicity [8,19,83,84,85].

Immune failure may arise from several barriers. First, effector T-cell recruitment may be inadequate when chemokine patterns favor suppressive myeloid cells, regulatory populations, or non-cytotoxic inflammation. Second, even when lymphocytes are present, they may remain spatially separated from tumor cells by fibroblast-rich stroma, dense extracellular matrix, abnormal vasculature, mesothelial-derived barriers, or spheroid architecture. Thus, peritoneal disease creates both fixed and mobile forms of immune exclusion across surfaces, implants, ascites, and tumor aggregates [23,26,29,85,86]. third barrier is functional persistence. T cells entering the peritoneal compartment are exposed to inhibitory cytokines, checkpoint ligands, nutrient competition, lactate, adenosine, hypoxia-related stress, extracellular vesicles, and chronic antigenic stimulation. These conditions can reduce proliferation, cytokine production, cytotoxicity, and metabolic fitness. Immune failure in the peritoneal cavity is therefore not only checkpoint-mediated, but also shaped by metabolic restriction and competition among tumor cells, macrophages, fibroblasts, and other stromal populations [4,8,19,29,87].

Antigen presentation may also be compromised. The peritoneal cavity can contain tumor antigens released by cell death, spheroid turnover, or tissue injury, but antigen availability does not guarantee productive immunity. Dendritic cells may be scarce, immature, or functionally inhibited, while macrophages may process tumor material in a tolerogenic rather than immunogenic context. If antigen is encountered in a milieu dominated by wound-healing signals, suppressive cytokines, and stromal remodeling, the result may be tolerance or dysfunction rather than T-cell priming [4,19].

Regulatory T cells and suppressive lymphoid states can further reinforce immune failure by limiting effector T-cell activation, inhibiting antigen-presenting cells, and stabilizing local tolerance. Natural killer cells may also be impaired by ascitic suppressive factors, reducing early clearance of free tumor cells and spheroids before lesions become fully stromalized [8,19,88,89,90].

### 5.5. Malignant Ascites as a Liquid Metastatic Microenvironment

**Figure 4:**
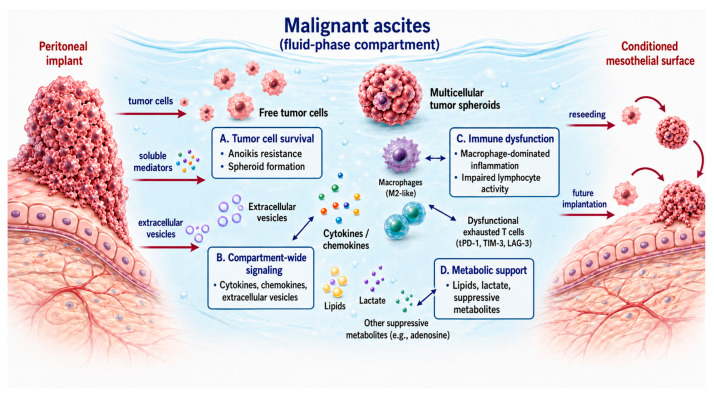
Malignant ascites as a liquid metastatic microenvironment. Malignant ascites functions as an active fluid-phase compartment in gastric cancer peritoneal metastasis. It contains free tumor cells, multicellular spheroids, extracellular vesicles, cytokines, chemokines, metabolites, macrophages, and dysfunctional lymphocytes. Through these components, ascites supports tumor cell survival, enables compartment-wide signaling, promotes immune dysfunction, provides metabolic support, and facilitates reseeding of conditioned peritoneal surfaces.

Malignant ascites is one of the defining features of advanced gastric cancer peritoneal metastasis. Clinically, it is associated with abdominal distension, impaired oral intake, reduced mobility, repeated procedures, and poor prognosis. Biologically, it functions as a liquid metastatic microenvironment in which tumor cells, spheroids, immune cells, cytokines, chemokines, extracellular vesicles, metabolites, matrix fragments, and therapeutic agents interact under conditions that differ from both the primary gastric tumor and attached peritoneal implants. By allowing malignant cells to persist in suspension, interact with host-derived cells, and reseed distant surfaces, ascites transforms peritoneal metastasis into a compartment-wide process [4,26,29,91]. The main biological functions of malignant ascites as a fluid-phase metastatic compartment are summarized in Figure 4.

Tumor cells in ascites must remain viable without stable extracellular matrix attachment. Resistance to anoikis, cell–cell adhesion, stress-response programs, and spheroid formation are therefore central to fluid-phase survival. Spheroids may act as mobile metastatic units that protect tumor cells from immune and therapeutic stress, preserve stem-like or quiescent states, and increase the probability of mesothelial adhesion and reseeding [23,29,79,92,93].

Ascitic fluid is also a reservoir of soluble and vesicle-mediated communication. Tumor cells, macrophages, mesothelial cells, fibroblasts, adipose-associated cells, and lymphocytes release cytokines, chemokines, growth factors, metabolites, matrix fragments, and extracellular vesicles into the fluid. These signals can promote mesothelial activation, fibroblast remodeling, macrophage polarization, immune suppression, spheroid survival, and drug resistance across anatomically distant peritoneal surfaces [4,29,91].

The immune and metabolic composition of ascites often favors tumor persistence rather than elimination. Macrophages, lymphocytes, neutrophils, dendritic cells, natural killer cells, and tumor cells may coexist in the fluid, but suppressive myeloid states, impaired lymphocyte function, lactate, lipids, amino acid derivatives, adenosine, hypoxia-associated factors, and oxidative stress can jointly support immune dysfunction, angiogenesis, fibrosis, and tumor survival [15,22,29,87].

The relationship between ascites and attached lesions is bidirectional. Peritoneal implants shed tumor cells, extracellular vesicles, cytokines, and matrix fragments into the fluid, while ascitic spheroids can reseed peritoneal surfaces and generate new implants. This exchange creates a feedback loop between solid and liquid disease compartments [29,91].

Ascites also offers an opportunity for repeated monitoring. Cytology, cell blocks, flow cytometry, single-cell sequencing, extracellular vesicle profiling, cytokine analysis, metabolomics, and functional testing may provide information about tumor viability, immune state, spheroid biology, and treatment response. However, ascites should be interpreted alongside tissue-based assessment because fluid cells may not fully represent stromal implants or spatial immune exclusion [29,55,91].

### 5.6. Metabolic Adaptation and Adipose-Associated Support in the Peritoneal Niche

Peritoneal metastasis requires substantial metabolic flexibility. Gastric cancer cells leaving the primary tumor may persist as free cells, form spheroids in ascites, attach to mesothelial surfaces, invade submesothelial stroma, or expand within adipose-rich regions such as the omentum. These states impose pressures that are not fully represented by the primary gastric tumor, including loss of matrix attachment, oxygen and nutrient gradients within spheroids, restricted diffusion in fibrotic implants, abnormal vascularization, and compartment-wide metabolic exchange through ascites [4,23,29,65].

Spheroid formation creates a small metabolic ecosystem within ascites. Peripheral cells may have greater access to oxygen and nutrients, whereas inner cells may become hypoxic, quiescent, or stress-resistant. This heterogeneity may contribute to drug tolerance and allow spheroids to serve as reservoirs for reseeding after therapy [26,79].

The omentum and mesenteric fat provide another major metabolic advantage. These adipose-rich structures are frequent sites of peritoneal implantation and cannot be viewed only as passive anatomical traps. Adipocytes can release fatty acids, adipokines, inflammatory mediators, and extracellular vesicles that influence tumor cell survival and phenotype. Tumor cells may induce lipolysis, take up fatty acids, and use lipid metabolism to support energy production, membrane synthesis, redox balance, or survival under stress. This adipose–tumor metabolic coupling may be particularly relevant in the peritoneal cavity, where glucose availability, oxygenation, and vascular access can be variable [65,78].

Adipose-associated stromal cells further expand the supportive role of these sites. They can provide growth factors, differentiate into fibroblast-like populations, produce extracellular matrix, and interact with macrophages and lymphocytes. In the omentum, metabolic, stromal, and immune support are closely linked: adipocytes supply energy-rich substrates, stromal cells stabilize implants, and macrophages shape inflammatory and immunosuppressive responses. This integrated niche may help explain why omental involvement is common in peritoneal carcinomatosis [4,18].

Hypoxia has particular relevance in peritoneal metastasis. It may develop within spheroids, poorly vascularized surface implants, dense stromal deposits, or ascites-associated aggregates. Hypoxia-inducible programs can enhance survival, angiogenesis, invasion, metabolic switching, and resistance to therapy. They may also increase vascular permeability and contribute to ascites formation. In established lesions, hypoxia and matrix remodeling can reinforce one another: dense extracellular matrix limits diffusion, while hypoxic signaling promotes fibroblast activation, angiogenesis, and further matrix deposition [4,26,93].

## 6. The Transition from Primary Tumor to Established Peritoneal Disease

Peritoneal metastasis in gastric cancer should be understood as a progressive transition rather than as a single event. Tumor cells may reach the peritoneal cavity through serosal invasion, exfoliation, or local tissue disruption, but dissemination and colonization are not equivalent. Clinically relevant disease emerges only when tumor cells survive loss of attachment, form free-floating aggregates or spheroids, adhere to activated mesothelium, invade the submesothelial compartment, and acquire stromal, vascular, metabolic, and immune support. These stages explain why peritoneal disease may remain biologically active but radiologically occult before becoming clinically dominant [4,23,27,94,95].

As lesions mature, attached implants and fluid-phase disease begin to reinforce one another. Implants shed tumor cells, cytokines, extracellular vesicles, and matrix fragments into ascites, while ascitic tumor cells and spheroids can reseed additional surfaces. This feedback converts peritoneal metastasis from a set of isolated deposits into a compartmental disease sustained by stromal remodeling, ascites-mediated signaling, immune dysfunction, and metabolic adaptation [4,26,29,32].

This distinction is relevant for clinical classification. Patients with positive peritoneal cytology, limited peritoneal implants, diffuse carcinomatosis, and massive ascites are often grouped under the broad label of peritoneal metastasis. Biologically, however, these states may differ. Positive cytology may indicate dissemination without established stromal disease. Limited implants may indicate successful adhesion and early anchoring. Ascites-rich carcinomatosis may indicate a mature compartmental process. Recognizing these differences could improve patient stratification, trial design, and selection of peritoneal-directed therapies [3,96]. The major biological states along the transition from primary tumor-derived dissemination to established peritoneal disease are summarized in Table 1.

The same logic applies to sampling. Peritoneal lavage, ascites, cytology cell blocks, omental biopsies, and peritoneal implants do not provide interchangeable information. They sample different phases of disease. Lavage may capture free-cell dissemination or early peritoneal exposure. Ascites reflects fluid-phase survival, immune suppression, and soluble communication. Peritoneal biopsies reveal stromal anchoring, matrix architecture, and immune exclusion. Omental lesions highlight adipose-associated and stromal-metabolic support. A comprehensive understanding of peritoneal metastasis requires aligning the sample type with the biological state being interrogated [29,32,96].

This framework also reframes the comparison with the primary tumor. The primary lesion creates the possibility of dissemination by generating invasive, stress-tolerant, and immune-evasive tumor cell states. The peritoneal compartment determines whether these states become clinically consequential. The same biological processes may therefore serve different purposes across sites. In the primary tumor, stromal remodeling facilitates invasion; in the peritoneum, it supports implantation. In the primary tumor, immune escape permits local expansion; in the peritoneum, immune failure permits survival across fluid and surface compartments. In the primary tumor, metabolic adaptation supports growth within abnormal tissue; in the peritoneum, it supports suspension survival, spheroid persistence, and adipose-associated growth [4,65,93].

In summary, gastric cancer peritoneal metastasis develops through a stepwise transition from primary tumor-derived dissemination to established compartmental disease. This perspective explains why exfoliation is not equivalent to metastasis, why peritoneal disease may remain occult before becoming clinically dominant, and why primary tumor biomarkers are informative but incomplete. Understanding these transitional states is essential for developing therapies that prevent implantation, eradicate minimal residual peritoneal disease, or disrupt the mature niche that sustains established carcinomatosis [4,93,96].

## 7. Therapeutic Implications of Microenvironmental Divergence

**Figure 5:**
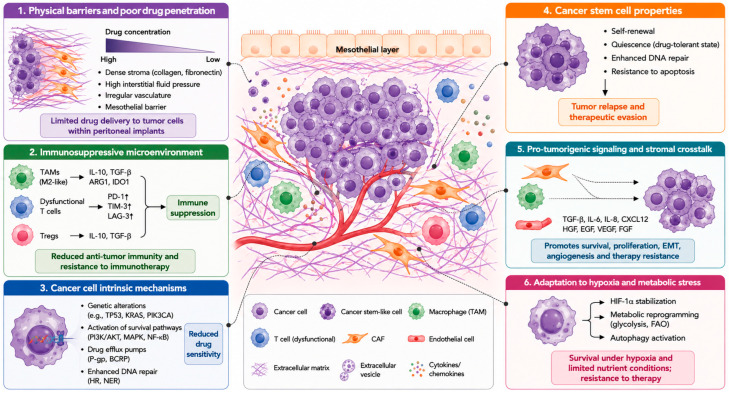
Mechanisms of therapeutic resistance in gastric cancer peritoneal metastasis. Therapeutic resistance in peritoneal metastasis results from the combined effects of tumor-intrinsic adaptation and microenvironmental protection. Dense extracellular matrix, irregular vascularization, and mesothelial or stromal barriers can limit drug delivery to peritoneal implants. At the same time, macrophage-dominated inflammation, dysfunctional lymphocytes, stromal cross-talk, hypoxia, metabolic stress, and spheroid-associated or stem-like tumor states can reduce sensitivity to systemic, locoregional, targeted, and immune-based therapies. These overlapping mechanisms support tumor persistence and may contribute to recurrence after treatment.

The therapeutic implications discussed in this section should be interpreted according to the maturity of the available evidence. Standard systemic therapy remains the main established treatment backbone for most patients with advanced gastric cancer and peritoneal metastasis, whereas peritoneal-directed strategies, niche-informed combinations, biomarker-guided compartmental sampling, and imaging- or radiomics-based approaches remain variably supported by clinical, translational, or hypothesis-generating evidence. Therefore, these concepts are presented as potential directions for patient stratification, trial design, and translational research rather than as established standards of care, unless explicitly stated otherwise. The biological divergence between the primary gastric tumor and peritoneal metastasis has direct therapeutic consequences. Peritoneal disease is difficult to treat not only because it represents advanced cancer, but because the compartment in which it develops modifies several determinants of response: drug delivery, stromal protection, immune accessibility, tumor cell state, spheroid architecture, ascites-mediated signaling, and metabolic adaptation. These factors help explain why systemic therapy often provides limited control of peritoneal metastasis and why treatment selection based only on primary tumor features may be insufficient [4]. The main microenvironmental mechanisms that contribute to therapeutic resistance in gastric cancer peritoneal metastasis are summarized in Figure 5.

A central therapeutic problem is access. Most systemic treatments rely on vascular delivery, whereas peritoneal metastases often develop as small, superficial, multifocal, and stromal-rich deposits. Some lesions are poorly vascularized or embedded within fibrotic tissue; others exist as free-floating tumor cells or spheroids in ascites. These disease forms are unlikely to experience drug exposure in the same way as a vascularized primary tumor or a hematogenous metastasis. Effective plasma drug concentration does not necessarily translate into effective peritoneal tumor exposure [4,32,58].

This problem extends beyond penetration into macroscopic nodules. Peritoneal disease is distributed across surfaces and fluid. A systemic agent must reach attached implants through abnormal vasculature, enter the peritoneal fluid compartment, penetrate spheroids, and remain active in the presence of soluble survival signals. Each step can reduce efficacy. This may partly explain why peritoneal progression can occur despite apparent control of disease at other sites [29].

Ascites further complicates pharmacology. It can dilute locally administered drugs, alter distribution and clearance, and provide tumor-protective signals. Tumor spheroids suspended in ascites may be shielded by cell–cell adhesion, extracellular matrix-like deposits, hypoxia gradients, and quiescent inner cell populations. Even direct exposure to drug-containing fluid may therefore fail to eliminate all viable tumor cells. The fluid phase of disease should be considered a therapeutic compartment, not simply a clinical symptom.

Locoregional therapies are attractive because they attempt to address this compartment directly. Intraperitoneal chemotherapy, hyperthermic intraperitoneal chemotherapy, pressurized intraperitoneal aerosol chemotherapy, and other peritoneal-directed approaches are based on the premise that peritoneal metastasis requires regional treatment. However, local delivery does not eliminate biological barriers. Drug distribution may remain heterogeneous, penetration into tumor nodules may be limited, and dense matrix or spheroid architecture may protect tumor cells. The success of locoregional therapy depends not only on where the drug is delivered, but on the state of the peritoneal niche at the time of treatment [58,93,97,98].

Timing is therefore critical. Peritoneal-directed strategies may be more effective in microscopic, low-volume, or cytology-positive disease, before mature stromal architecture, high-volume ascites, and extensive immune suppression are established. In advanced carcinomatosis, the challenge is different: therapy must overcome fibrotic implants, abnormal vasculature, ascitic tumor reservoirs, and compartment-wide immunosuppression. Preventing implantation and treating mature peritoneal disease are biologically distinct goals and should not be approached as if they were the same clinical problem [4,18,58].

The stromal compartment is a major mediator of therapeutic resistance. Fibroblasts and extracellular matrix can limit drug penetration, provide survival signals, alter tumor metabolism, and restrict immune cell access. In peritoneal metastasis, this protection is especially relevant because tumor cells require stromal anchoring to convert surface attachment into durable growth. Once established, this scaffold can shield tumor cells from both systemic and intraperitoneal treatment.

For this reason, stromal modulation may be more realistic than stromal elimination. The aim would be to reduce excessive matrix protection, improve drug penetration, normalize aspects of vascular function, disrupt tumor–matrix survival signaling, or increase immune accessibility. Such approaches may be particularly valuable in combination with chemotherapy, targeted therapy, or immunotherapy. However, stromal targeting requires caution, because some stromal responses may reflect host attempts to contain disease or repair tissue injury. The therapeutic goal should be to remodel a protective niche, not indiscriminately remove all stromal components [4,18,58].

The myeloid compartment represents another therapeutic target. Macrophages and related populations contribute to stromal remodeling, angiogenesis, ascites formation, spheroid survival, and suppression of adaptive immunity. In the peritoneal cavity, their influence extends across both attached lesions and fluid compartments. Strategies that block monocyte recruitment, inhibit macrophage survival signals, reprogram suppressive macrophage states, or disrupt macrophage–fibroblast communication could weaken the niche that sustains peritoneal disease. As with stroma, functional reprogramming may be preferable to broad depletion, given the homeostatic and antimicrobial roles of peritoneal macrophages [26,71,88].

These considerations are particularly relevant to immunotherapy. Immune checkpoint inhibition depends on the presence of tumor-reactive lymphocytes that can enter lesions, persist, and function. In peritoneal metastasis, each of these requirements may be compromised. Effector cells may be poorly recruited, trapped at stromal margins, suppressed by macrophages, metabolically impaired by ascites, or unable to penetrate spheroids. Therefore, checkpoint blockade may fail even when tumor-intrinsic markers suggest potential immunogenicity [90].

Rational immunotherapy combinations should address the dominant immune barrier in the peritoneal compartment. In some patients, the limiting factor may be insufficient effector T-cell recruitment. In others, it may be stromal exclusion, macrophage-dominated suppression, defective antigen presentation, regulatory lymphocyte enrichment, or metabolic inhibition. Peritoneal-specific immune profiling may help identify which barrier is most relevant. The therapeutic question is not only whether the tumor can be recognized, but whether the peritoneal niche permits immune execution.

Metabolic adaptation also creates therapeutic vulnerabilities. Peritoneal tumor cells may rely on lipid utilization, hypoxia-response programs, spheroid-associated stress tolerance, lactate signaling, and metabolic exchange with adipose-rich structures. These dependencies may not be evident from primary tumor profiling alone. Targeting fatty acid uptake or oxidation, lactate-mediated suppression, hypoxia adaptation, or spheroid survival pathways could theoretically weaken peritoneal disease. However, metabolic strategies require careful validation because many pathways are shared by tumor and normal host cells [29,87,93].

Ascites-directed therapy deserves particular consideration. Current management is often palliative, focused on drainage and symptom relief. A microenvironmental perspective suggests that ascites should also be viewed as a biological target. It contains viable tumor cells, spheroids, suppressive immune populations, extracellular vesicles, cytokines, metabolites, and factors that condition mesothelium and stroma. Therapies that reduce ascites formation, eliminate tumor spheroids, neutralize soluble survival signals, or restore immune function within the fluid compartment may reduce reseeding and improve disease control [26,29,58,98].

Targeted therapy must also be interpreted through the peritoneal niche. Expression of HER2, CLDN18.2, FGFR2b, MET, or other targets is commonly assessed in primary tumor tissue, but peritoneal response may depend on target preservation in metastatic lesions, tissue penetration, stromal shielding, spheroid architecture, immune effector mechanisms, and compensatory survival signals. A lesion may express a target yet remain protected by poor access or microenvironmental rescue. Conversely, peritoneal lesions may acquire dependencies not prominent in the sampled primary tumor. When feasible, metastatic or ascitic sampling may therefore improve therapeutic interpretation.

Antibody-based therapies and antibody–drug conjugates may be especially sensitive to microenvironmental context. Their activity depends not only on antigen expression, but also on delivery, tissue penetration, internalization, bystander effects, and interaction with immune effector cells. In a macrophage-rich, lymphocyte-poor, matrix-dense peritoneal niche, these variables may differ from those in the primary tumor. Understanding why such therapies succeed or fail in peritoneal disease will require analysis of both tumor target expression and niche architecture [32,99].

The same logic applies to cellular therapies and regional immune approaches. The peritoneal cavity is anatomically accessible, making intraperitoneal delivery attractive. However, infused cells must persist in a hostile environment characterized by suppressive cytokines, hypoxia, nutrient competition, ascites, macrophage dominance, and stromal barriers. Successful cellular therapy may therefore require engineering or combination strategies that improve trafficking, persistence, resistance to suppression, and activity against both spheroids and attached implants [90,100,101].

Therapeutic strategy should also distinguish prevention of peritoneal recurrence from treatment of established carcinomatosis. In high-risk patients with serosal invasion, positive cytology, or minimal peritoneal disease, the goal may be to eliminate free cells, prevent spheroid formation, block adhesion, or interrupt early niche conditioning. In established ascites-rich carcinomatosis, the goal shifts toward disrupting mature stromal and immune support, controlling the fluid reservoir, and improving drug penetration. These are different biological states and should be reflected in treatment design [3,58].

Surgery and cytoreductive approaches should also be interpreted through this lens. Cytoreduction reduces macroscopic burden, but recurrence may arise from microscopic implants, residual spheroids, or a conditioned peritoneal environment. Completeness of cytoreduction is therefore not only a surgical measure; it reflects the extent to which the visible disease component has been removed. Durable benefit may require combining cytoreduction with therapies that address residual fluid-phase disease and niche-mediated survival [3,58,96].

Clinical trial design should better reflect peritoneal biology. Patients with positive cytology only, limited implants, and diffuse ascites-rich carcinomatosis should not be treated as a homogeneous population. Stratification by peritoneal cancer index, ascites status, cytology, prior treatment, resectability, and locoregional therapy exposure may improve interpretation. Correlative studies should include peritoneal compartment samples whenever feasible, rather than relying exclusively on archival primary tumor tissue.

Endpoints should also be adapted. Overall survival remains essential, but peritoneal disease produces clinically meaningful effects that may not be captured by conventional response criteria. Cytology conversion, ascites control, paracentesis-free interval, peritoneal cancer index reduction, time to bowel obstruction, nutritional stabilization, symptom burden, and quality of life may all reflect therapeutic benefit. These endpoints should not be viewed as secondary details; they correspond to the biological and clinical behavior of the peritoneal compartment [29,58,96,102].

Treatment-induced niche remodeling is another important consideration. Chemotherapy may reduce tumor burden but also promote inflammation, fibrosis, macrophage recruitment, or stromal repair. Anti-angiogenic therapy may alter vascular permeability and ascites but may also modify hypoxia. Immunotherapy may activate lymphocytes without overcoming stromal exclusion or macrophage suppression. Intraperitoneal therapy may reduce superficial disease while leaving protected spheroids or fibrotic implants. Understanding these adaptive responses may help explain relapse and guide sequencing [4,29,93].

In summary, the therapeutic challenge of gastric cancer peritoneal metastasis is not limited to the malignant epithelial cell. The peritoneal niche modifies drug exposure, protects tumor cells, suppresses immunity, supports metabolic adaptation, and permits recurrence through fluid-phase reseeding. Effective treatment will likely require stage-adapted and compartment-aware strategies that combine tumor-directed therapy with approaches targeting stromal protection, myeloid suppression, ascites biology, metabolic dependencies, and drug delivery barriers. This shift from tumor-only treatment to niche-informed therapy is essential for improving outcomes in peritoneal disease [4,26,29,93].

### 7.1. Biomarker Development: Moving Beyond the Primary Tumor Sample

Biomarker development in gastric cancer peritoneal metastasis is limited by a fundamental sampling problem: the tissue most often analyzed is not always the tissue that determines the clinical course. In routine practice, biomarkers are frequently assessed in diagnostic biopsies or surgical specimens from the primary gastric tumor. This approach is practical and remains essential for histological and molecular classification, but it may not capture the biological state of established peritoneal disease. When progression is driven by ascites, microscopic implants, stromal-protected lesions, or immune suppression within the peritoneal cavity, primary tumor profiling provides only part of the relevant information [4,29,32].

The goal should therefore not be to replace primary tumor biomarkers, but to complement them with compartment-specific markers. The primary tumor defines the origin of the disease and provides critical information on histology, molecular subtype, mismatch repair status, HER2 expression, PD-L1 expression, CLDN18.2 expression, and other therapeutic targets. Peritoneal samples, by contrast, can reveal whether disseminated cells have acquired fluid-phase survival programs, whether the mesothelial surface is conditioned for implantation, whether metastases are stromal-rich or immune-excluded, and whether ascites contains suppressive or tumor-supportive mediators. These are different biological questions and require different sampling strategies [32,35,96]. To summarize these practical differences, Table 2 compares the main clinical and translational implications of primary tumor–peritoneal niche divergence.

In practical terms, peritoneal compartment assessment may be most informative in selected clinical or translational contexts: when peritoneal disease is the dominant site of progression, when treatment response differs between primary or visceral lesions and peritoneal disease, when ascites is clinically significant and accessible for cytology or cell-block preparation, or when enrollment in biomarker-driven or peritoneal-directed clinical trials is being considered. Peritoneal biopsy, ascites analysis, lavage cytology, and cytology cell blocks should therefore be viewed as complementary to primary tumor profiling rather than as routine replacements for it. At present, established clinical biomarkers in gastric cancer remain primarily tumor-centered, including HER2, MSI/MMR status, PD-L1 CPS, and, where available, CLDN18.2 or other approved target markers. By contrast, ascites-derived immune signatures, spatial immune organization, fibroblast subsets, mesothelial activation markers, spheroid phenotypes, extracellular vesicles, and radiomics-based features remain investigational and require prospective validation before routine clinical use. A peritoneal-specific biomarker framework should integrate several specimen types: peritoneal implants, omental lesions, ascites, peritoneal lavage, cytology cell blocks, and, when informative, circulating tumor DNA. Each captures a distinct layer of disease. Tissue biopsies reveal architecture, matrix organization, vascular proximity, immune exclusion, and tumor–stroma interactions. Ascites captures the fluid phase, including free tumor cells, spheroids, immune populations, extracellular vesicles, soluble mediators, and metabolites. Lavage may detect occult dissemination or early peritoneal exposure before macroscopic disease develops. Cytology cell blocks may provide tumor material when tissue biopsy is not feasible. None of these samples is complete alone; their value lies in their complementarity [29,96,103].

Ascites is particularly useful because it can sometimes be sampled repeatedly. Serial analysis may show whether therapy reduces viable tumor cells, disrupts spheroids, modifies macrophage or lymphocyte populations, changes cytokine patterns, or alters metabolic suppression. This longitudinal potential is important in peritoneal metastasis, where radiological assessment is often insensitive and tissue access may be limited. Ascites can therefore function as a dynamic window into the peritoneal compartment [29,96,103].

However, ascites should not be treated as a perfect surrogate for attached lesions. Its composition is influenced by tumor burden, vascular permeability, lymphatic obstruction, inflammation, nutritional status, prior therapy, and sampling conditions. Tumor cells suspended in fluid may differ from tumor cells embedded in stromal implants. Lymphocytes in ascites may not reflect lymphocytes excluded from tumor nests. Soluble mediators may originate from tumor cells, macrophages, mesothelial cells, fibroblasts, adipose tissue, or systemic inflammation. Ascites-based biomarkers must therefore be interpreted in relation to tissue findings and clinical context [29,32,96].

Peritoneal lavage offers a different opportunity, particularly in patients without visible peritoneal disease. Conventional cytology can identify free intraperitoneal cancer cells, but it provides limited biological information. More informative approaches could assess tumor-derived nucleic acids, epithelial markers, extracellular vesicle cargo, immune mediators, mesothelial activation signals, or early stromal conditioning. Such markers may help distinguish transient tumor cell shedding from biologically meaningful peritoneal colonization risk. This distinction is clinically important because the presence of free cells alone does not necessarily indicate that the peritoneal niche is permissive [26,103,104].

Cytology-positive disease deserves special consideration. It occupies an intermediate state between localized gastric cancer and established peritoneal metastasis. It indicates that tumor cells have reached the peritoneal cavity, but not necessarily that stromal implants, mature ascites, or compartment-wide immune suppression are present. Biomarkers in this setting should therefore focus on early survival and implantation potential: anoikis resistance, spheroid formation, mesothelial adhesion programs, immune evasion in fluid, and signs of peritoneal conditioning. Treating cytology-positive disease as biologically identical to diffuse carcinomatosis risks missing an important therapeutic window [3,105].

Solid peritoneal biopsies remain essential because they provide spatial information that fluid cannot capture. The density and orientation of extracellular matrix, the localization of macrophages, the proximity of cytotoxic lymphocytes to tumor cells, the presence of mesothelial-derived stromal changes, and the organization of vascular structures all require tissue context. A lesion may contain immune cells but remain functionally immune-excluded if those cells are confined to stromal margins. Similarly, fibroblast abundance is less informative than fibroblast position, matrix architecture, and relationship to tumor nests.

For this reason, spatial biomarkers may be especially valuable in peritoneal metastasis. Relevant features include tumor–lymphocyte distance, stromal thickness, collagen organization, macrophage localization at tumor–stroma interfaces, vascular proximity, and distribution of mesothelial or fibroblast-like populations. These features cannot be inferred reliably from bulk expression data. A transcriptomic signature may suggest inflammation, but spatial analysis is needed to determine whether that inflammation is tumor-directed or trapped outside malignant regions [4,18,29,32].

Spheroid biology should also be incorporated into biomarker development. Tumor aggregates in ascites may represent a clinically relevant reservoir of disease and a source of reseeding. Useful markers may include cell–cell adhesion programs, extracellular matrix incorporation, hypoxia-related features, stemness-associated states, immune shielding, and drug penetration characteristics. Spheroid-rich ascites may identify patients in whom fluid-phase disease is a major driver of recurrence or treatment resistance [4,29,93].

Immune biomarkers require peritoneal-specific interpretation. In the primary tumor, PD-L1 expression, mismatch repair deficiency, Epstein–Barr virus status, tumor mutational burden, and immune infiltration may inform immunotherapy decisions. In peritoneal disease, these markers should be complemented by measures of immune accessibility and function: effector-to-regulatory T-cell balance, macrophage-to-lymphocyte ratio, dendritic cell maturity, NK-cell activity, T-cell exhaustion, suppressive cytokines, metabolic inhibition, and spatial immune exclusion. A peritoneal metastasis may be antigenic but inaccessible, infiltrated but dysfunctional, or inflamed but dominated by non-protective myeloid programs [8,19,32,69].

Stromal and matrix biomarkers are equally important. Fibroblast activation, collagen density, matrix alignment, integrin signaling, mesothelial-to-mesenchymal-like changes, angiogenic patterns, and stromal thickness may influence progression and treatment response. A matrix-dense implant may require different therapeutic strategies from a spheroid-dominant fluid phenotype or a lesion with loose inflammatory stroma. These distinctions are unlikely to be captured by primary tumor biomarkers alone [18,26].

Metabolic biomarkers may provide an additional layer of stratification. Ascitic lactate, lipid composition, hypoxia-associated signatures, adipokines, amino acid depletion, adenosine-related pathways, and fatty acid utilization programs could identify patients whose disease is driven by metabolic adaptation to ascites, adipose-rich surfaces, or hypoxic stromal implants. Such markers may be particularly informative when integrated with immune profiling, because metabolic suppression can strongly influence T-cell and macrophage function [65,87].

Temporal heterogeneity must also be considered. Biomarkers measured at diagnosis may not reflect the state of peritoneal disease after chemotherapy, surgery, intraperitoneal therapy, or immunotherapy. Treatment can alter antigen expression, ascites composition, immune cell states, fibroblast activation, extracellular vesicle cargo, and spheroid viability. Static biomarkers may therefore be less useful than longitudinal markers that track the evolution of the peritoneal compartment under therapy [26,29,103].

Minimal residual peritoneal disease is a particularly important setting. After gastrectomy or systemic treatment, patients may harbor free tumor cells, microscopic implants, or conditioned peritoneal surfaces that are not detectable by imaging. Biomarkers capable of identifying residual viable tumor cells, early stromal anchoring, mesothelial activation, or persistent ascitic survival signals could improve risk stratification and guide adjuvant or locoregional interventions. This may be one of the settings in which peritoneal-directed biomarkers have the greatest clinical value [96].

A useful biomarker should also be biologically actionable. It should not only correlate with prognosis, but indicate a mechanism that can be targeted or monitored. A macrophage-dominant ascitic profile may support myeloid-modulating strategies. A matrix-dense peritoneal implant may suggest stromal modulation or improved drug delivery. A spheroid-rich fluid phenotype may support approaches targeting aggregation, anoikis resistance, or intraperitoneal exposure. A metabolically suppressive ascites profile may indicate immune-metabolic vulnerability. Biomarkers should therefore help classify peritoneal disease according to dominant biological barriers [26,29,32].

For clinical trials, correlative studies should avoid exclusive reliance on archival primary tumor tissue. Whenever feasible, trials enrolling patients with peritoneal metastasis should incorporate baseline and on-treatment ascites, lavage, cytology cell blocks, or peritoneal biopsies. Stratification by peritoneal disease state—positive cytology only, limited implants, high-volume carcinomatosis, ascites-dominant disease—may reduce biological heterogeneity and improve interpretation of treatment effects [96,103].

In summary, biomarker development for gastric cancer peritoneal metastasis must move beyond exclusive dependence on the primary tumor sample. Primary tumor profiling remains essential, but it does not fully describe fluid-phase survival, mesothelial interaction, stromal anchoring, immune exclusion, ascites-mediated signaling, or metabolic adaptation. A clinically useful biomarker strategy should integrate primary tumor features with peritoneal compartment sampling and spatially aware interpretation. Only then can biomarkers reflect the disease state that most directly determines progression and therapeutic resistance [29,32,103,105].

### 7.2. Response Assessment in Peritoneal Metastasis: Why Conventional Endpoints Are Insufficient

Assessing response in gastric cancer peritoneal metastasis is challenging because the disease is often biologically active before it becomes easily measurable. Conventional response criteria were developed primarily for solid lesions that can be followed by changes in diameter on imaging. Peritoneal metastasis does not consistently follow this pattern. It may manifest as positive cytology, microscopic implants, diffuse serosal thickening, small nodules, omental infiltration, bowel tethering, malignant ascites, or functional gastrointestinal impairment. Many of these features are poorly captured by standard radiological measurements.

This limitation reflects the biology of peritoneal disease. Unlike hematogenous metastases, which often form discrete lesions within vascularized organs, peritoneal metastasis is distributed across surfaces and fluid. A patient may have viable tumor cells in ascites, spheroids, conditioned mesothelial surfaces, and early stromal implants without large measurable masses. Conversely, a therapy may reduce ascitic tumor burden, convert cytology to negative, improve bowel function, or control ascites without producing a conventional radiological response. If assessment relies only on lesion size, clinically meaningful effects may be missed [58,96,106,107].

Radiological evaluation is particularly limited in early or low-volume peritoneal disease. Small implants on bowel serosa, mesentery, diaphragmatic peritoneum, or pelvic surfaces can be below the resolution of computed tomography. Diffuse infiltration may be subtle or nonspecific, especially after surgery or systemic therapy. Ascites may suggest peritoneal involvement, but it does not directly quantify viable tumor burden. Larger nodules and omental caking are easier to detect, but they often represent more advanced disease. Imaging therefore tends to identify established peritoneal metastasis more reliably than the earlier stages when intervention may be most effective.

This creates a mismatch between biological progression and radiological progression. Peritoneal recurrence may begin with free tumor cells, spheroid formation, microscopic implantation, or early mesothelial and stromal conditioning. These processes can precede visible disease. Similarly, resistance may develop within ascitic spheroids or stromal-protected microscopic deposits before measurable progression is documented. Radiology often captures the consequence of peritoneal progression rather than the process itself [3,96,108].

Cytology provides a complementary but incomplete measure. Positive peritoneal cytology indicates that tumor cells have reached the peritoneal cavity and is clinically meaningful even in the absence of visible implants. Conversion from positive to negative cytology may reflect therapeutic effect and could serve as an important endpoint in selected settings. However, cytology depends on sampling quality, tumor cell shedding, assay sensitivity, and interpretation. A negative result does not exclude microscopic implants, and a positive result does not distinguish transient free cells from cells capable of implantation. Cytology should therefore be interpreted as one layer of compartmental assessment, not as a complete measure of disease status [3,96].

Molecular analysis of lavage fluid may improve sensitivity and provide information beyond morphology. Detection of tumor-derived nucleic acids, epithelial markers, extracellular vesicles, or methylation patterns could help identify occult peritoneal disease. However, molecular positivity also requires careful interpretation. Tumor-derived material may originate from viable cells, dying cells, extracellular vesicles, or residual debris after treatment. Its clinical meaning may depend on timing, assay type, and whether the signal persists longitudinally. Molecular lavage markers are likely to be most useful when combined with cytology, imaging, clinical risk factors, and serial monitoring [103].

Ascites control is another clinically relevant endpoint. In patients with malignant ascites, reduction in fluid accumulation can improve abdominal distension, appetite, mobility, respiratory comfort, nutritional intake, and treatment tolerance. Biologically, ascites reduction may indicate decreased vascular permeability, reduced tumor activity, improved lymphatic drainage, or disruption of the fluid-phase niche. However, ascites volume alone is not a precise surrogate for tumor response. It can be influenced by paracentesis, albumin status, portal pressure, inflammation, hydration, and supportive care. More informative assessment would combine ascites volume with paracentesis frequency, cytology, tumor cell viability, immune composition, and symptom improvement [58,91,106].

The peritoneal cancer index provides a more anatomically specific measure of macroscopic peritoneal burden, particularly in surgical or laparoscopic contexts. It captures lesion distribution and size across peritoneal regions and is useful for selecting patients for cytoreductive or locoregional approaches. Its limitations are that it is invasive, not easily repeated, and primarily reflects visible disease. A low peritoneal cancer index may coexist with positive cytology or biologically active ascites, whereas a high index may not capture the immune, stromal, or metabolic state of the disease. It is therefore valuable, but not sufficient as a standalone biological endpoint [96,106].

Symptom-based and functional endpoints are especially important in peritoneal metastasis. The disease often harms patients through bowel dysfunction, impaired motility, early satiety, nausea, abdominal pain, ascites-related discomfort, malnutrition, and reduced performance status. A therapy that delays bowel obstruction, reduces paracentesis frequency, stabilizes nutrition, or improves oral intake may provide meaningful benefit even if measurable tumor shrinkage is modest. These outcomes should not be viewed as merely palliative secondary measures; they reflect the clinical consequences of peritoneal compartment control [4,91].

Nutritional and functional measures may also help capture disease behavior. Weight loss, sarcopenia, hypoalbuminemia, inflammatory markers, and performance status are nonspecific, but they often reflect the combined effects of tumor burden, ascites, bowel involvement, systemic inflammation, and metabolic dysregulation. When interpreted together with imaging, cytology, ascites parameters, and symptoms, these measures can provide a more clinically relevant picture of response than imaging alone [91,109,110].

For clinical trials, the limitations of conventional response assessment have major consequences. Patients with diffuse peritoneal disease may be excluded from trials because they lack measurable lesions. Therapies that act on ascites, cytology, spheroids, microscopic implants, or symptom burden may appear inactive if judged only by standard radiological response. Conversely, stable measurable lesions may coexist with worsening ascites, bowel symptoms, or cytology. Trial endpoints should therefore be aligned with the biology and clinical behavior of peritoneal metastasis [106,107].

A more appropriate response framework would combine several layers of assessment. Imaging remains necessary, but should be complemented by cytology or molecular lavage analysis, ascites evaluation, peritoneal cancer index when feasible, symptom measures, paracentesis frequency, nutritional status, and longitudinal biological sampling. The relative importance of each endpoint depends on disease state. In cytology-positive disease without visible implants, cytology conversion and molecular clearance may be most relevant. In limited macroscopic disease, peritoneal burden and completeness of cytoreduction may matter more. In ascites-dominant carcinomatosis, ascites control, fluid tumor burden, symptom relief, and quality of life may be central [96,103,107].

Longitudinal assessment is particularly important. A single time point may be misleading because peritoneal disease fluctuates with fluid dynamics, treatment effects, inflammation, and procedures. Serial evaluation of ascites, cytology, molecular markers, symptoms, and nutritional status may reveal trends that are more informative than isolated measurements. Decreasing viable tumor cells in ascites may precede radiological improvement, whereas rising tumor-derived markers in lavage or ascites may signal early progression before imaging becomes positive [29,55].

Imaging methods may also need refinement. Diffusion-weighted MRI, selected PET-based approaches, radiomics, and artificial intelligence-supported image analysis may improve detection or characterization of peritoneal disease, although each has limitations. The goal is not only to identify more lesions, but to better capture distribution, cellularity, fibrosis, perfusion, and treatment-induced change. Even with improved imaging, biological sampling will remain important because many relevant features of peritoneal metastasis are cellular, immune, molecular, or fluid-based rather than purely anatomical [29,107,111].

Distinguishing treatment effect from progression can be difficult. Therapy may induce inflammation, fibrosis, necrosis, changes in vascular permeability, or fluctuations in ascites. Residual fibrotic implants may persist after tumor cell death, while stable-appearing peritoneal thickening may contain viable resistant disease. Increased ascites may reflect progression, inflammation, or altered vascular permeability depending on context. Fluid and tissue biomarkers may help resolve these ambiguities by identifying viable tumor cells, immune activation, stromal remodeling, or molecular residual disease [32,103].

Response assessment after immunotherapy requires particular caution. Apparent changes in peritoneal thickening or ascites may reflect progression, immune infiltration, inflammation, or vascular effects. A stable lesion size may conceal shifts in immune composition or tumor viability. Immunotherapy trials in peritoneal disease should therefore incorporate immune monitoring of ascites and tissue whenever possible. Without such data, it is difficult to determine whether treatment failed because the tumor was non-immunogenic, because lymphocytes were excluded, or because the peritoneal niche remained suppressive [19,90,112].

For locoregional therapies, response assessment should capture both local cytotoxic effect and compartmental control. After intraperitoneal chemotherapy, HIPEC, PIPAC, or related approaches, relevant endpoints may include cytology conversion, histological regression in peritoneal biopsies, reduction in peritoneal cancer index, ascites control, symptom improvement, and time to peritoneal progression. Histological response can be informative, but sampling error remains a concern because peritoneal disease is heterogeneous. Standardized biopsy sites or repeated sampling may improve interpretability [107,113].

Minimal residual peritoneal disease is an emerging assessment challenge. After surgery or systemic therapy, residual tumor burden may persist as isolated cells, spheroids, microscopic implants, or conditioned niche components. These may not be visible on imaging but can drive relapse. Sensitive detection methods in lavage or ascites may help identify residual disease, but the field must determine which signals are clinically meaningful and actionable. The ideal minimal residual disease marker would identify viable, metastasis-competent cells or a peritoneal niche state strongly associated with recurrence [96,103,114].

Ultimately, response assessment in peritoneal metastasis should move from lesion-based evaluation toward compartment-based evaluation. This does not mean abandoning radiology or survival endpoints. Rather, it means recognizing that peritoneal disease behaves as a surface–fluid process. A complete assessment should ask whether therapy reduces viable tumor cells, clears cytology, disrupts spheroids, controls ascites, improves immune function, limits stromal implantation, preserves gastrointestinal function, and delays compartmental failure [107,115,116].

In summary, conventional response criteria are insufficient for gastric cancer peritoneal metastasis because the disease is diffuse, surface-based, fluid-associated, biologically heterogeneous, and often poorly measurable. Effective assessment requires integration of imaging, cytology, lavage or ascites biomarkers, peritoneal burden scores, symptom endpoints, nutritional measures, and longitudinal sampling. Such a framework would better reflect the biology of peritoneal disease and improve evaluation of therapies designed to target both tumor cells and the metastatic niche that sustains them [32,96,107].

## 8. Future Directions: Studying Peritoneal Metastasis as a Site-Specific Disease State

Future progress in gastric cancer peritoneal metastasis will depend on studying the peritoneal compartment directly rather than inferring its biology from the primary tumor alone. The primary lesion remains essential for diagnosis and baseline molecular classification, but peritoneal disease introduces biological questions that require site-specific investigation: how tumor cells survive in suspension, how they interact with mesothelial surfaces, how stromal support is assembled, how ascites sustains reseeding, and why local immunity often fails despite inflammatory activity.

A first priority is systematic paired sampling. Studies limited to primary tumors can identify features associated with risk of peritoneal relapse, but they cannot fully define established peritoneal disease. Conversely, studies focused only on advanced carcinomatosis may miss early events that permit dissemination and implantation. The most informative designs would integrate primary tumor tissue, invasive or serosa-adjacent regions, peritoneal lavage, ascites, peritoneal implants, omental lesions, and samples collected before and after treatment. Such approaches would help distinguish tumor programs inherited from the primary lesion from adaptations induced by the peritoneal niche [9,29,86].

This distinction is essential. A feature enriched in peritoneal metastasis may represent a preexisting aggressive clone, a response to ascitic or mesothelial cues, a consequence of stromal selection, an effect of prior therapy, or a combination of these. Without temporal and paired sampling, these possibilities cannot be separated. Future studies should therefore treat peritoneal metastasis as a dynamic process, moving from occult dissemination to microscopic implantation, established implants, ascites-rich disease, and therapy-exposed residual disease [9,117].

Single-cell technologies can help define the cellular states involved in this transition. Peritoneal metastasis includes malignant epithelial cells, mesothelial cells, fibroblasts, macrophages, lymphocytes, endothelial cells, adipose-associated stromal cells, and tumor cells suspended in ascites. Single-cell RNA sequencing, immune profiling, and multiomic methods can identify tumor cell plasticity, stromal heterogeneity, immune dysfunction, and compartment-specific adaptation. These tools are particularly valuable for detecting rare or transitional populations that may be missed by bulk profiling [29,118].

However, dissociated single-cell data must be complemented by spatial methods. Peritoneal metastasis is organized around surfaces, interfaces, and barriers. The biological meaning of a cell state depends on where it is located: whether lymphocytes contact tumor cells or remain trapped in stroma, whether macrophages cluster around spheroids or invasive fronts, whether fibroblasts form exclusionary matrix layers, and whether mesothelial cells remain intact or become incorporated into metastatic stroma. Spatial transcriptomics, multiplex immunofluorescence, imaging mass cytometry, and related technologies are therefore essential for understanding the architecture of peritoneal immune exclusion and stromal protection [9,18,19].

Ascites profiling should become a central component of future research. Malignant ascites provides access to tumor cells, spheroids, immune populations, extracellular vesicles, cytokines, metabolites, and therapy-induced changes. Because it can sometimes be sampled repeatedly, ascites offers an opportunity to monitor the evolving peritoneal ecosystem over time. Longitudinal ascites analysis could reveal whether treatment reduces viable spheroids, shifts macrophage or lymphocyte states, changes metabolic suppression, or alters extracellular vesicle-mediated signaling [26,29,103].

Peritoneal lavage is equally important in earlier disease. In patients at high risk of peritoneal recurrence, lavage may reveal free tumor cells or molecular signals before macroscopic metastases develop. Future studies should determine whether lavage-based markers can distinguish transient tumor cell shedding from biologically meaningful peritoneal colonization risk. This will likely require combining tumor-cell detection with markers of spheroid formation, mesothelial activation, immune suppression, extracellular vesicle cargo, and early stromal conditioning [29,103].

Better preclinical models are also needed. Conventional two-dimensional gastric cancer cell lines cannot reproduce the critical steps of peritoneal dissemination. Models should incorporate suspension survival, spheroid formation, mesothelial adhesion, stromal interaction, ascites-like fluid conditions, immune components, and adipose-associated support. Patient-derived organoids, ascites-derived spheroids, mesothelial co-cultures, fibroblast and macrophage co-cultures, microfluidic systems, ex vivo peritoneal explants, and orthotopic or intraperitoneal animal models each address different aspects of the disease. The model should be matched to the biological question [26,93].

For example, spheroid and fluid-phase models are appropriate for studying anoikis resistance, drug penetration into aggregates, and ascites-mediated survival. Mesothelial co-cultures or peritoneal explants are better suited for adhesion and implantation. Immune-competent models are required to study macrophage–lymphocyte interactions and immunotherapy. Matrix-rich three-dimensional systems are necessary for drug penetration and stromal protection. Future work should avoid using convenient models to answer biologically mismatched questions.

Treatment pressure should also be incorporated into experimental design. Many clinical samples are obtained after chemotherapy, surgery, intraperitoneal therapy, or immunotherapy. Treatment can reshape tumor cell states, antigen expression, fibroblast activation, macrophage programs, ascites composition, and spheroid viability. Therapy-exposed models and longitudinal patient samples may therefore be more informative than untreated systems alone when investigating resistance [26,29,93].

Another important direction is the development of peritoneal-specific disease classifications. Current gastric cancer classifications are largely derived from primary tumor biology. While valuable, they do not capture the dominant niche states that may drive peritoneal progression. A peritoneal classification could integrate tumor cell phenotype, ascites composition, immune balance, stromal architecture, mesothelial activation, metabolic features, and disease distribution. Such a classification should not replace existing molecular subtypes, but should add a compartmental layer relevant to prognosis and therapy selection [19,32].

For this to be clinically useful, classification must remain actionable. A macrophage-dominant, lymphocyte-poor ascitic phenotype may suggest myeloid-modulating strategies. A matrix-dense, immune-excluded implant phenotype may require stromal modulation or improved drug delivery. A spheroid-rich fluid phenotype may call for approaches targeting aggregation, anoikis resistance, or intraperitoneal exposure. A metabolically lipid-adapted phenotype may suggest investigation of tumor–adipose metabolic coupling. These categories remain to be validated, but they illustrate how peritoneal-specific profiling could guide rational therapeutic hypotheses [19,32].

Clinical trials should also be designed around the biology of peritoneal disease. Patients with positive cytology only, limited implants, and diffuse ascites-rich carcinomatosis should not automatically be treated as a single homogeneous group. Trials should stratify or report outcomes according to peritoneal burden, ascites status, cytology, prior therapy, resectability, and locoregional treatment exposure. Correlative studies should include peritoneal compartment samples whenever feasible rather than relying only on archival primary tumor tissue.

Endpoints should likewise reflect peritoneal behavior. Overall survival remains essential, but intermediate endpoints should include cytology conversion, ascites control, paracentesis-free interval, peritoneal cancer index change, histological regression in peritoneal biopsies, time to bowel obstruction, nutritional stabilization, symptom burden, and quality of life. These endpoints are not peripheral in peritoneal metastasis; they capture the ways in which the disease progresses and affects patients [107].

Minimal residual peritoneal disease may be one of the most important future settings. After gastrectomy or systemic therapy, residual disease may persist as free cells, spheroids, microscopic implants, or conditioned peritoneal surfaces that are not visible on imaging. Detecting and treating this state may be more effective than treating established carcinomatosis. Future studies should define reliable markers of minimal residual peritoneal disease and test interventions designed to eliminate free cells, prevent implantation, or reverse early niche conditioning.

Computational integration may help manage the complexity of these datasets. Imaging, histology, spatial profiling, single-cell data, ascites cytokines, metabolomics, and clinical outcomes will need to be analyzed together. Radiomics and artificial intelligence-supported image analysis may improve detection and quantification of subtle peritoneal disease, while multimodal models may identify recurrent patterns of niche organization. These tools will be useful only if trained on well-annotated datasets with peritoneal-specific endpoints [32,103,114,119].

Finally, future research should remain connected to patient-centered outcomes. Peritoneal metastasis causes morbidity through ascites, bowel dysfunction, nutritional decline, pain, repeated procedures, and loss of functional status. Molecular findings should therefore be linked not only to survival, but also to ascites formation, bowel obstruction, cachexia, treatment tolerance, and quality of life. A biologically sophisticated model of peritoneal metastasis is valuable only if it improves the ability to prevent, monitor, or treat the clinical syndrome patients experience.

In summary, gastric cancer peritoneal metastasis should be studied as a site-specific disease state. Progress will require paired and longitudinal sampling, integration of tissue and fluid compartments, single-cell and spatial technologies, biologically appropriate models, peritoneal-specific biomarkers, and trials with endpoints matched to the behavior of the compartment. The goal is not only to describe how peritoneal metastasis differs from the primary tumor, but to identify which differences can be targeted, monitored, and translated into better outcomes [107,109,115].

## 9. Limitations

This review has several limitations. First, as a narrative review, it is subject to selection bias and does not provide a systematic or quantitative synthesis of the literature. Second, the available evidence is heterogeneous, combining clinical studies, translational analyses, preclinical models, and mechanistic data derived from different experimental systems. Third, direct evidence from gastric cancer peritoneal metastasis remains limited for some biological domains, particularly mesothelial remodeling, ascites-mediated immune modulation, spatial immune organization, and niche-directed therapeutic targeting. In these areas, some concepts are supported by preclinical studies or extrapolated from other peritoneal malignancies. Finally, many proposed biomarkers and microenvironmental features discussed in this review remain investigational and require prospective validation before they can be incorporated into routine clinical decision-making.

## 10. Conclusions

Peritoneal metastasis in gastric cancer illustrates why metastatic site matters biologically. Although peritoneal disease originates from the primary gastric tumor, it cannot be understood as a simple anatomical extension of it. The transition from the gastric wall to the peritoneal cavity exposes tumor cells to a different set of constraints: survival outside tissue architecture, interaction with mesothelial surfaces, adaptation to fluid-phase conditions, acquisition of stromal support, and evasion of local immune control. These constraints reshape both malignant cells and host tissues, creating a metastatic niche that differs from the primary tumor in organization, function, and therapeutic vulnerability.

The primary tumor remains essential to this process. It generates cellular diversity, selects invasive and stress-tolerant phenotypes, and provides the initial immune and stromal context from which dissemination may emerge. However, the peritoneal compartment determines whether disseminated cells become clinically consequential. Tumor cells that reach the peritoneal cavity must survive in suspension, form aggregates, implant on permissive surfaces, and integrate into a stromal and immune environment that supports persistence. Peritoneal carcinomatosis is therefore not the inevitable result of tumor cell shedding, but the outcome of successful adaptation to a specialized host compartment.

This distinction has practical implications. Biomarkers derived from the primary tumor define important aspects of tumor identity, but they may not capture the dominant biological barriers operating in peritoneal disease. Likewise, therapies selected on the basis of primary tumor features may fail if response is limited by poor drug penetration, stromal protection, macrophage-dominated immune suppression, ascites-mediated survival, spheroid architecture, or metabolic adaptation. The therapeutic challenge is therefore not only to target malignant epithelial cells, but also to disrupt the peritoneal niche that sustains them.

Future progress will require direct study of the peritoneal compartment. Ascites, peritoneal lavage, cytology specimens, peritoneal implants, and omental lesions provide complementary information that cannot be replaced by primary tumor analysis alone. Integrating these samples with spatial profiling, single-cell technologies, immune characterization, metabolic analysis, and clinically relevant endpoints may allow more accurate stratification and more rational treatment development.

In conclusion, gastric cancer peritoneal metastasis should be regarded as a site-specific biological state shaped by both tumor cell adaptation and microenvironmental reprogramming. The primary tumor explains where dissemination begins; the peritoneal niche explains why the disease becomes so difficult to control. Recognizing this distinction is essential for developing biomarkers, response measures, and therapeutic strategies that address not only the cancer cell, but the compartment that allows it to persist.

## Figures and Tables

**Table 1:** Peritoneal disease states in gastric cancer: biological features, informative samples, and translational implications.

Peritoneal Disease State	Dominant Biological Process	Key Microenvironmental Features	Most Informative Samples	Main Translational Implication
High-risk primary tumor without detectable peritoneal disease	Generation of dissemination-competent tumor states	Serosal proximity, invasive-front remodeling, immune–stromal selection, matrix remodeling, tumor cell plasticity	Primary tumor biopsy/surgical specimen, invasive front, serosa-adjacent tissue	Primary tumor profiling may identify risk, but does not prove peritoneal colonization
Positive peritoneal cytology/occult free tumor cells	Intraperitoneal dissemination without established macroscopic implants	Free tumor cells, early anoikis resistance, possible spheroid initiation, exposure to peritoneal immune surveillance	Peritoneal lavage, cytology cell block, molecular lavage assays	Represents a potential therapeutic window before stable stromal anchoring
Free-floating spheroid-dominant disease	Fluid-phase survival and reseeding potential	Multicellular aggregates, cell–cell survival signaling, partial immune shielding, ascitic soluble factors, extracellular vesicles	Ascites, cytology cell block, single-cell ascites profiling, spheroid assays	Therapies should address spheroid survival, anoikis resistance, and fluid-phase tumor reservoirs
Early microscopic implantation	Adhesion to mesothelium and access to submesothelial matrix	Mesothelial activation, junctional loosening, basement membrane exposure, early macrophage/fibroblast recruitment	Peritoneal lavage, targeted peritoneal biopsy if feasible, spatial tissue analysis	Anti-adhesion, mesothelial-conditioning, or early niche-interruption strategies may be most relevant
Limited macroscopic peritoneal implants	Stromal anchoring and early metastatic outgrowth	CAF-like stromal support, ECM deposition, macrophage recruitment, emerging immune exclusion, abnormal vascular adaptation	Peritoneal implant biopsy, omental biopsy, PCI assessment, spatial profiling	Disease may still be suitable for locoregional or combined systemic–regional strategies
Ascites-dominant carcinomatosis	Compartment-wide fluid–surface disease	Malignant ascites, tumor spheroids, macrophage-rich inflammation, dysfunctional lymphocytes, soluble cytokines/EVs/metabolites, reseeding	Ascites, cytology cell block, immune/metabolic profiling, serial sampling	Ascites should be treated as a biological disease compartment, not only as a symptom
Fibrotic/stromal-rich carcinomatosis	Mature niche protection and therapeutic insulation	Dense ECM, stromal barriers, poor vascularization, immune exclusion, hypoxia, limited drug penetration	Peritoneal biopsy, omental biopsy, spatial ECM/immune analysis, imaging/PCI	Therapeutic failure may be driven by niche protection rather than tumor target absence
Post-treatment residual peritoneal disease	Survival of protected microscopic or fluid-phase reservoirs	Residual spheroids, microscopic implants, treatment-induced fibrosis, persistent suppressive immune states, molecular residual disease	Serial ascites/lavage, cytology, molecular residual disease assays, repeat biopsy when feasible	Requires longitudinal monitoring; radiological stability may underestimate biological persistence

**Table 2:** Practical comparison of primary tumor and peritoneal niche features, highlighting evidence type, clinical implications, and therapeutic relevance in gastric cancer peritoneal metastasis.

Biological Domain	Primary Tumor Feature	Peritoneal Niche Feature	Evidence Type	Clinical Implication	Therapeutic Relevance
Anatomical context	Organ-embedded tumor within the gastric wall	Surface-based implants and fluid-phase disease within the peritoneal cavity	Clinical and translational	Primary tumor biology may not fully represent the compartment driving symptoms and progression	Supports site-specific interpretation of disease behavior and treatment response
Tumor cell state	Invasive tumor cells shaped by gastric wall stroma, immune pressure, and local hypoxia	Detached cells, multicellular spheroids, and implanted tumor cells adapted to suspension, adhesion, and mesothelial invasion	Translational and preclinical	Peritoneal disease may include non-measurable fluid-phase tumor populations in addition to solid implants	Therapies may need activity against both attached implants and free-floating tumor aggregates
Mesothelial interaction	Usually absent from routine primary tumor assessment	Tumor adhesion to activated or disrupted mesothelium, followed by submesothelial invasion	Mainly preclinical and translational	Peritoneal biopsy may capture implantation biology not inferred from primary tumor sampling	Mesothelial adhesion and invasion pathways remain investigational targets
Stromal and ECM remodeling	Desmoplasia, fibroblast activation, and matrix remodeling at invasive fronts	Submesothelial stromal anchoring, fibrosis, matrix remodeling, and barriers to drug penetration	Translational and preclinical	Stromal-rich peritoneal lesions may resist therapy despite actionable primary tumor biomarkers	Supports investigation of stromal modulation and improved intraperitoneal drug delivery
Immune microenvironment	Variable immune infiltration, immune exclusion, tertiary lymphoid structures, or PD-L1 expression in primary tissue	Myeloid-dominant inflammation, T-cell dysfunction, immune exclusion, and ascites-associated immunosuppression	Clinical and translational	Primary tumor immune markers may not fully reflect the immune state of peritoneal disease	May help explain limited immunotherapy efficacy in some patients with peritoneal metastasis
Malignant ascites	No equivalent fluid compartment in the primary gastric tumor	Tumor cells, spheroids, immune cells, cytokines, extracellular vesicles, metabolites, and stromal mediators in ascitic fluid	Clinical, translational, and preclinical	Ascites cytology, cell blocks, and molecular analysis may provide additional biological information when feasible	Ascites-mediated survival and resistance remain important research and therapeutic targets
Biomarker expression	HER2, PD-L1, MSI/MMR, CLDN18.2, FGFR2b, MET, and other markers are commonly assessed in primary tissue	Biomarker expression may differ between primary tumor, peritoneal implants, and ascites-derived tumor cells	Clinical and translational	Re-biopsy or ascites-based testing may be useful in selected patients with progression or discordant response	May refine targeted therapy selection, but prospective validation is needed
Drug accessibility	Systemic therapy reaches the tumor through vascularized gastric wall tissue	Peritoneal implants may have limited vascularization, stromal shielding, spheroid architecture, and ascites-mediated dilution or sequestration	Clinical, translational, and preclinical	Lack of response may reflect compartmental barriers, not only intrinsic tumor resistance	Supports investigation of intraperitoneal delivery approaches, while avoiding overstatement of established benefit
Response assessment	Primary or visceral lesions may be measurable by conventional imaging	Diffuse implants, ascites, bowel dysfunction, nutritional decline, and positive cytology may be poorly captured by RECIST	Clinical	Imaging alone may underestimate peritoneal disease burden or progression	Supports integrating imaging with symptoms, ascites dynamics, cytology, and functional status
Translational modeling	Primary tumor tissue, bulk molecular profiling, and tumor-derived organoids are commonly used	Peritoneal biopsies, ascites-derived spheroids, co-culture systems, single-cell analysis, and spatial profiling may better model peritoneal disease	Translational and preclinical	Models should reflect both fluid-phase and surface-based disease	Useful for studying niche-mediated resistance and testing peritoneal-directed strategies

## Data Availability

No new data were created or analyzed in this study. Data sharing is not applicable to this article.

## References

[1] Bray F., Laversanne M., Sung H., Ferlay J., Siegel R.L., Soerjomataram I., Jemal A. (2024). Global Cancer Statistics 2022: GLOBOCAN Estimates of Incidence and Mortality Worldwide for 36 Cancers in 185 Countries. CA Cancer J. Clin..

[2] Rijken A., Lurvink R.J., Luyer M.D.P., Nieuwenhuijzen G.A.P., van Erning F.N., van Sandick J.W., de Hingh I.H.J.T. (2021). The Burden of Peritoneal Metastases from Gastric Cancer: A Systematic Review on the Incidence, Risk Factors and Survival. J. Clin. Med..

[3] Manzanedo I., Pereira F., Pérez-Viejo E., Serrano Á. (2023). Gastric Cancer with Peritoneal Metastases: Current Status and Prospects for Treatment. Cancers.

[4] Ng D., Cyr D., Khan S., Dossa F., Swallow C., Kazazian K. (2025). Molecular Mechanisms of Metastatic Peritoneal Dissemination in Gastric Adenocarcinoma. Cancer Metastasis Rev..

[5] Yonemura Y., Ishibashi H., Mizumoto A., Tukiyama G., Liu Y., Wakama S., Sako S., Takao N., Kitai T., Katayama K. (2022). The Development of Peritoneal Metastasis from Gastric Cancer and Rationale of Treatment According to the Mechanism. J. Clin. Med..

[6] Sun F., Feng M., Guan W. (2017). Mechanisms of Peritoneal Dissemination in Gastric Cancer. Oncol. Lett..

[7] Li J., Guo T. (2022). Role of Peritoneal Mesothelial Cells in the Progression of Peritoneal Metastases. Cancers.

[8] Takahashi K., Kurashina K., Yamaguchi H., Kanamaru R., Ohzawa H., Miyato H., Saito S., Hosoya Y., Lefor A.K., Sata N. (2022). Altered Intraperitoneal Immune Microenvironment in Patients with Peritoneal Metastases from Gastric Cancer. Front. Immunol..

[9] Wang R., Song S., Harada K., Ghazanfari Amlashi F., Badgwell B., Pizzi M.P., Xu Y., Zhao W., Dong X., Jin J. (2020). Multiplex Profiling of Peritoneal Metastases from Gastric Adenocarcinoma Identified Novel Targets and Molecular Subtypes That Predict Treatment Response. Gut.

[10] Chen Y., Cai G., Jiang J., He C., Chen Y., Ding Y., Lu J., Zhao W., Yang Y., Zhang Y. (2023). Proteomic Profiling of Gastric Cancer with Peritoneal Metastasis Identifies a Protein Signature Associated with Immune Microenvironment and Patient Outcome. Gastric Cancer.

[11] Peng H., Jiang L., Yuan J., Wu X., Chen N., Liu D., Liang Y., Xie Y., Jia K., Li Y. (2024). Single-Cell Characterization of Differentiation Trajectories and Drug Resistance Features in Gastric Cancer with Peritoneal Metastasis. Clin. Transl. Med..

[12] Fan Y., Vykoukal J.V., Song S., Pizzi M.P., Zou G., Yoshimura K., Jin J., Katayama H., Calin G.A., Waters R.E. (2025). Protocol for Isolating Patient-Derived Ascites Cells and Extracellular Vesicles from Gastric Cancer Peritoneal Metastases. STAR Protoc..

[13] Zheng L.-N., Wen F., Xu P., Zhang S. (2019). Prognostic Significance of Malignant Ascites in Gastric Cancer Patients with Peritoneal Metastasis: A Systemic Review and Meta-Analysis. World J. Clin. Cases.

[14] Jiao Y., Peng X., Wang Y., Hao Z., Chen L., Wu M., Zhang Y., Li J., Li W., Zhan X. (2023). Malignant Ascites Supernatant Enhances the Proliferation of Gastric Cancer Cells Partially via the Upregulation of Asparagine Synthetase. Oncol. Lett..

[15] Song H., Wang T., Tian L., Bai S., Chen L., Zuo Y., Xue Y. (2019). Macrophages on the Peritoneum Are Involved in Gastric Cancer Peritoneal Metastasis. J. Cancer.

[16] Raal F.J., Stein E.A., Dufour R., Turner T., Civeira F., Burgess L., Langslet G., Scott R., Olsson A.G., Sullivan D. (2015). PCSK9 Inhibition with Evolocumab (AMG 145) in Heterozygous Familial Hypercholesterolaemia (RUTHERFORD-2): A Randomised, Double-Blind, Placebo-Controlled Trial. Lancet.

[17] Murphy S.A., Pedersen T.R., Gaciong Z.A., Ceska R., Ezhov M.V., Connolly D.L., Jukema J.W., Toth K., Tikkanen M.J., Im K. (2019). Effect of the PCSK9 Inhibitor Evolocumab on Total Cardiovascular Events in Patients With Cardiovascular Disease: A Prespecified Analysis From the FOURIER Trial. JAMA Cardiol..

[18] Zhao J.J., Ong C.-A.J., Srivastava S., Chia D.K.A., Ma H., Huang K., Sheng T., Ramnarayanan K., Ong X., Tay S.T. (2024). Spatially resolved niche and tumor microenvironmental alterations in gastric cancer peritoneal metastases. Gastroenterology.

[19] Kim H., Kwon M., Lee S.K., Son S.-M., Lee O.-J., Man Yoon S., Kim H.K., Yang Y., Lee K.H., Han H.S. (2025). Distinct Immunosuppressive Tumor Microenvironment in Gastric Cancer With Peritoneal Metastasis. J. Gastric Cancer.

[20] Saiz Martínez R., Dromain C., Vietti Violi N. (2021). Imaging of Gastric Carcinomatosis. J. Clin. Med..

[21] Hamamoto Y. (2015). Complications in Advanced or Recurrent Gastric Cancer Patients with Peritoneal Metastasis during and after Palliative Systemic Chemotherapy. Mol. Clin. Oncol..

[22] Nakanishi T., Imano M., Kohda M., Kato H., Kounami N., Yamada A., Terada M., Hiraki Y., Shiraishi O., Yasuda A. (2025). Intraperitoneal Immune Microenvironment and Efficacy of Intraperitoneal Chemotherapy in Patients with Gastric Cancer and Peritoneal Metastasis. Sci. Rep..

[23] Kang D., Kim I.-H. (2022). Molecular Mechanisms and Potential Rationale of Immunotherapy in Peritoneal Metastasis of Advanced Gastric Cancer. Biomedicines.

[24] Wilson R.B., Solass W., Archid R., Weinreich F.-J., Königsrainer A., Reymond M.A. (2019). Resistance to Anoikis in Transcoelomic Shedding: The Role of Glycolytic Enzymes. Pleura Peritoneum.

[25] Al Habyan S., Kalos C., Szymborski J., McCaffrey L. (2018). Multicellular Detachment Generates Metastatic Spheroids during Intra-Abdominal Dissemination in Epithelial Ovarian Cancer. Oncogene.

[26] Zhang X., Zhang C., Zhang Z., Zhang X., Wang K. (2026). Modeling the Pre-Metastatic Niche of Gastric Cancer Peritoneal Metastasis under Spatiotemporal Resolution and Investigating EVs-Mediated Immune Suppression. Front. Immunol..

[27] Ren K., Xie X., Min T., Sun T., Wang H., Zhang Y., Dang C., Zhang H. (2022). Development of the Peritoneal Metastasis: A Review of Back-Grounds, Mechanisms, Treatments and Prospects. J. Clin. Med..

[28] Matsuoka T., Yashiro M. (2024). Molecular Mechanism for Malignant Progression of Gastric Cancer Within the Tumor Microenvironment. Int. J. Mol. Sci..

[29] Huang X.-Z., Pang M.-J., Li J.-Y., Chen H.-Y., Sun J.-X., Song Y.-X., Ni H.-J., Ye S.-Y., Bai S., Li T.-H. (2023). Single-Cell Sequencing of Ascites Fluid Illustrates Heterogeneity and Therapy-Induced Evolution during Gastric Cancer Peritoneal Metastasis. Nat. Commun..

[30] Ramos C., Gerakopoulos V., Oehler R. (2024). Metastasis-Associated Fibroblasts in Peritoneal Surface Malignancies. Br. J. Cancer.

[31] Satala C.B., Bara T.J., Jung I., Tudorache V., Gurzu S. (2021). Chylous Ascites, Unusual Association with Ductal Pancreatic Adenocarcinoma with Plasmacytoid Morphology: A Case Report and Literature Review. Surg. J..

[32] Sun W., Jiang Z., Deng Z. (2025). Comprehensive analysis of the tumor immune microenvironment in gastric cancer and peritoneal metastasis based on single-cell RNA sequencing analysis. Sci. Rep..

[33] Zavros Y., Merchant J.L. (2022). The Immune Microenvironment in Gastric Adenocarcinoma. Nat. Rev. Gastroenterol. Hepatol..

[34] Oya Y., Hayakawa Y., Koike K. (2020). Tumor Microenvironment in Gastric Cancers. Cancer Sci..

[35] Satala C.B., Jung I., Stefan-van Staden R.I., Kovacs Z., Molnar C., Bara T., Fulop Z.Z., Gurzu S. (2020). HER2 Heterogeneity in Gastric Cancer: A Comparative Study, Using Two Commercial Antibodies. J. Oncol..

[36] Lee S.H., Lee D., Choi J., Oh H.J., Ham I.-H., Ryu D., Lee S.-Y., Han D.-J., Kim S., Moon Y. (2025). Spatial Dissection of Tumour Microenvironments in Gastric Cancers Reveals the Immunosuppressive Crosstalk between CCL2+ Fibroblasts and STAT3-Activated Macrophages. Gut.

[37] Chen B., Tang H., Zheng X., Xie F., Yu P., Lyu Y., Feng T., Wu J., Liu J., Xu Y. (2025). Spatial and Functional Dissection of Cancer-Associated Fibroblasts-Mediated Immune Modulation in H. Pylori-Associated Gastric Cancer. Mol. Cancer.

[38] Ma M., Sun J., Liu Z., Ouyang S., Zhang Z., Zeng Z., Li J., Kang W. (2022). The Immune Microenvironment in Gastric Cancer: Prognostic Prediction. Front. Oncol..

[39] Satala C.-B., Gurau G., Gurau A.-M., Patrichi G., Mihalache D. (2026). Tumor Budding in Gastric Carcinoma: Beyond Counting Cells at the Invasive Front—A Review of Current Evidence and Biological Perspectives. Int. J. Mol. Sci..

[40] Jia X., Li Z., Zhou R., Feng W., Yi L., Zhang H., Chen B., Li Q., Huang S., Zhu X. (2024). Single Cell and Bulk RNA Sequencing Identifies Tumor Microenvironment Subtypes and Chemoresistance-Related IGF1+ Cancer-Associated Fibroblast in Gastric Cancer. Biochim. Biophys. Acta Mol. Basis Dis..

[41] Gao S., Qin S., Wang D., Wang A., Zhu L., Li Y., Shi Q., Fan H., Bo Y., Zhong Y. (2026). A Spatially Resolved Atlas of Gastric Cancer Characterises a Lymphocyte-Aggregated Region. Nat. Commun..

[42] Wang Y., Zhang G., Zhang X., Liu G., Zhang L., Chen L., Sang S., Yao S., Fei Y., Tian Z. (2025). Single-Cell and Spatial Transcriptomics Implicate a Prognostic Function of Tertiary Lymphoid Structures in Gastric Cancer. Nat. Commun..

[43] Yang S., Wei S., Wei F. (2024). Extracellular Vesicles Mediated Gastric Cancer Immune Response: Tumor Cell Death or Immune Escape?. Cell Death Dis..

[44] Satala C.-B., Jung I., Kovacs Z., Stefan-Van Staden R.-I., Molnar C., Bara T., Patrichi A.-I., Gurzu S. (2022). V-Set and Immunoglobulin Domain Containing 1 (VSIG1) as an Emerging Target for Epithelial-Mesenchymal Transition of Gastric Cancer. Sci. Rep..

[45] Wang H., Yang L., Chen W., Li K., Xu M., Peng X., Li J., Zhao F., Wang B. (2024). High-Resolution Subtyping of Fibroblasts in Gastric Cancer Reveals Diversity among Fibroblast Subsets and an Association between the MFAP5-Fibroblast Subset and Immunotherapy. Front. Immunol..

[46] Satala C.-B., Patrichi G., Gurau A.-M., Onofrei Popa A., Mihalache D. (2026). Beyond Gastric Specificity: V-Set and Immunoglobulin Domain-Containing 1 (VSIG1) in Digestive Tract Tumors. Cancers.

[47] Qiu L., Zhao X., Yao S., Fei Y., Gong Y., Zhou Z., Jiao S., Xu J. (2025). Multi-Omics Analyses Reveal Interactions between GREM1+ Fibroblasts and SPP1+ Macrophages in Gastric Cancer. NPJ Precis. Oncol..

[48] Yun H., Dong F., Wei X., Yan X., Zhang R., Zhang X., Wang Y. (2025). Role and Value of the Tumor Microenvironment in the Progression and Treatment Resistance of Gastric Cancer (Review). Oncol. Rep..

[49] Moreira A.M., Pereira J., Melo S., Fernandes M.S., Carneiro P., Seruca R., Figueiredo J. (2020). The Extracellular Matrix: An Accomplice in Gastric Cancer Development and Progression. Cells.

[50] Chivu-Economescu M., Necula L.G., Matei L., Dragu D., Bleotu C., Sorop A., Herlea V., Dima S., Popescu I., Diaconu C.C. (2022). Collagen Family and Other Matrix Remodeling Proteins Identified by Bioinformatics Analysis as Hub Genes Involved in Gastric Cancer Progression and Prognosis. Int. J. Mol. Sci..

[51] Yasuda T., Wang Y.A. (2024). Gastric Cancer Immunosuppressive Microenvironment Heterogeneity: Implications for Therapy Development. Trends Cancer.

[52] Mou P., Ge Q., Sheng R., Zhu T., Liu Y., Ding K. (2023). Research Progress on the Immune Microenvironment and Immunotherapy in Gastric Cancer. Front. Immunol..

[53] Tang D., Liu S., Shen H., Deng G., Zeng S. (2022). Extracellular Vesicles Promote the Formation of Pre-Metastasis Niche in Gastric Cancer. Front. Immunol..

[54] Sato Y., Okamoto K., Kawano Y., Kasai A., Kawaguchi T., Sagawa T., Sogabe M., Miyamoto H., Takayama T. (2023). Novel Biomarkers of Gastric Cancer: Current Research and Future Perspectives. J. Clin. Med..

[55] Khoo B.L. (2023). Early Detection of Metastasis in Ascites and Peritoneal Lavage—New Perspectives Using Label-Free Microfluidic Approaches. eBioMedicine.

[56] Li Q., Li B., Li Q., Wei S., He Z., Huang X., Wang L., Xia Y., Xu Z., Li Z. (2018). Exosomal miR-21-5p Derived from Gastric Cancer Promotes Peritoneal Metastasis via Mesothelial-to-Mesenchymal Transition. Cell Death Dis..

[57] Sheng R., Yin Y., Wang X. (2025). Mesothelial and Immune Cells Interplay in the Tumor Microenvironment. Trends Mol. Med..

[58] Prabhu A., Mishra D., Brandl A., Yonemura Y. (2022). Gastric Cancer With Peritoneal Metastasis—A Comprehensive Review of Current Intraperitoneal Treatment Modalities. Front. Oncol..

[59] Kitayama J., Ishigami H., Yamaguchi H., Sakuma Y., Horie H., Hosoya Y., Lefor A.K., Sata N. (2018). Treatment of Patients with Peritoneal Metastases from Gastric Cancer. Ann. Gastroenterol. Surg..

[60] Cortés-Guiral D., Hübner M., Alyami M., Bhatt A., Ceelen W., Glehen O., Lordick F., Ramsay R., Sgarbura O., Van Der Speeten K. (2021). Primary and Metastatic Peritoneal Surface Malignancies. Nat. Rev. Dis. Primers.

[61] Bootsma S., Bijlsma M.F., Vermeulen L. (2023). The Molecular Biology of Peritoneal Metastatic Disease. EMBO Mol. Med..

[62] Hou S., Wang J., Li W., Hao X., Hang Q. (2021). Roles of Integrins in Gastrointestinal Cancer Metastasis. Front. Mol. Biosci..

[63] Winkler J., Abisoye-Ogunniyan A., Metcalf K.J., Werb Z. (2020). Concepts of Extracellular Matrix Remodelling in Tumour Progression and Metastasis. Nat. Commun..

[64] Raskov H., Orhan A., Gaggar S., Gögenur I. (2021). Cancer-Associated Fibroblasts and Tumor-Associated Macrophages in Cancer and Cancer Immunotherapy. Front. Oncol..

[65] Natsume M., Shimura T., Iwasaki H., Okuda Y., Hayashi K., Takahashi S., Kataoka H. (2020). Omental Adipocytes Promote Peritoneal Metastasis of Gastric Cancer through the CXCL2–VEGFA Axis. Br. J. Cancer.

[66] Kinoshita J., Doden K., Sakimura Y., Hayashi S., Saito H., Tsuji T., Yamamoto D., Moriyama H., Minamoto T., Inaki N. (2024). Crosstalk Between Omental Adipose-Derived Stem Cells and Gastric Cancer Cells Regulates Cancer Stemness and Chemotherapy Resistance. Cancers.

[67] Rietveld P.C.S., Guchelaar N.A.D., Sassen S.D.T., Koch B.C.P., Mathijssen R.H.J., Koolen S.L.W. (2025). A Clinical Pharmacological Perspective on Intraperitoneal Chemotherapy. Drugs.

[68] Satala C.-B., Jung I., Gurzu S. (2023). Mucin-Phenotype and Expression of the Protein V-Set and Immunoglobulin Domain Containing 1 (VSIG1): New Insights into Gastric Carcinogenesis. Int. J. Mol. Sci..

[69] Li Y., Zheng Y., Huang J., Nie R.-C., Wu Q.-N., Zuo Z., Yuan S., Yu K., Liang C.-C., Pan Y.-Q. (2024). CAF-Macrophage Crosstalk in Tumour Microenvironments Governs the Response to Immune Checkpoint Blockade in Gastric Cancer Peritoneal Metastases. Gut.

[70] Wang C., Fan X., Sun X., Xu Y., Sun Y., Liu J. (2025). Tumor associated macrophages in gastric cancer dual roles in immune evasion and clinical implications for targeted therapy. Front. Immunol..

[71] Che K., Luo Y., Song X., Yang Z., Wang H., Shi T., Wang Y., Wang X., Wu H., Yu L. (2024). Macrophages Reprogramming Improves Immunotherapy of IL-33 in Peritoneal Metastasis of Gastric Cancer. EMBO Mol. Med..

[72] He Y., Hong Q., Chen S., Zhou J., Qiu S. (2025). Reprogramming Tumor-Associated Macrophages in Gastric Cancer: A Pathway to Enhanced Immunotherapy. Front. Immunol..

[73] Gong Y., Yang J., Wang Y., Xue L., Wang J. (2020). Metabolic Factors Contribute to T-Cell Inhibition in the Ovarian Cancer Ascites. Int. J. Cancer.

[74] Pu Y., Ji Q. (2022). Tumor-Associated Macrophages Regulate PD-1/PD-L1 Immunosuppression. Front. Immunol..

[75] Saris J., Li Yim A.Y.F., Bootsma S., Lenos K.J., Franco Fernandez R., Khan H.N., Verhoeff J., Poel D., Mrzlikar N.M., Xiong L. (2025). Peritoneal Resident Macrophages Constitute an Immunosuppressive Environment in Peritoneal Metastasized Colorectal Cancer. Nat. Commun..

[76] Zhang Y., Ouyang D., Chen Y.H., Xia H. (2022). Peritoneal Resident Macrophages in Tumor Metastasis and Immunotherapy. Front. Cell Dev. Biol..

[77] Etzerodt A., Moulin M., Doktor T.K., Delfini M., Mossadegh-Keller N., Bajenoff M., Sieweke M.H., Moestrup S.K., Auphan-Anezin N., Lawrence T. (2020). Tissue-Resident Macrophages in Omentum Promote Metastatic Spread of Ovarian Cancer. J. Exp. Med..

[78] Hamabe-Horiike T., Harada S., Yoshida K., Kinoshita J., Yamaguchi T., Fushida S. (2023). Adipocytes Contribute to Tumor Progression and Invasion of Peritoneal Metastasis by Interacting with Gastric Cancer Cells as Cancer Associated Fibroblasts. Cancer Rep..

[79] D’Amore T., Bravoco D., Di Paola G., Albano F., Brancaccio M., Sabato C., Cesta G., Zolfanelli C., Lauciello V., Falco G. (2025). Anoikis Resistance in Gastric Cancer: A Comprehensive Review. Cell Death Dis..

[80] Ma C., Li Y., Li M., Lv C., Tian Y. (2025). Targeting immune checkpoints on myeloid cells: Current status and future directions. Cancer Immunol. Immunother..

[81] Liu Y., Xiao H., Zeng H., Xiang Y. (2024). Beyond Tumor-Associated Macrophages Involved in Spheroid Formation and Dissemination: Novel Insights for Ovarian Cancer Therapy (Review). Int. J. Oncol..

[82] Shoji H., Kudo-Saito C., Nagashima K., Imazeki H., Tsugaru K., Takahashi N., Kawakami T., Amanuma Y., Wakatsuki T., Okano N. (2024). Myeloid Subsets Impede the Efficacy of Anti-PD1 Therapy in Patients with Advanced Gastric Cancer (WJOG10417GTR Study). J. Immunother. Cancer.

[83] Eum H.H., Kwon M., Ryu D., Jo A., Chung W., Kim N., Hong Y., Son D.-S., Kim S.T., Lee J. (2020). Tumor-promoting macrophages prevail in malignant ascites of advanced gastric cancer. Exp. Mol. Med..

[84] Chen W., Zhang L., Gao M., Zhang N., Wang R., Liu Y., Niu Y., Jia L. (2025). Role of Tertiary Lymphoid Structures and B Cells in Clinical Immunotherapy of Gastric Cancer. Front. Immunol..

[85] Groen-van Schooten T.S., Franco Fernandez R., van Grieken N.C.T., Bos E.N., Seidel J., Saris J., Martínez-Ciarpaglini C., Fleitas T.C., Thommen D.S., de Gruijl T.D. (2024). Mapping the Complexity and Diversity of Tertiary Lymphoid Structures in Primary and Peritoneal Metastatic Gastric Cancer. J. Immunother. Cancer.

[86] Yonemura A., Semba T., Zhang J., Fan Y., Yasuda-Yoshihara N., Wang H., Uchihara T., Yasuda T., Nishimura A., Fu L. (2024). Mesothelial Cells with Mesenchymal Features Enhance Peritoneal Dissemination by Forming a Protumorigenic Microenvironment. Cell Rep..

[87] Cui M.-Y., Yi X., Zhu D.-X., Wu J. (2022). The Role of Lipid Metabolism in Gastric Cancer. Front. Oncol..

[88] Li Y., Jiang L., Chen Y., Li Y., Yuan J., Lu J., Zhang Z., Liu S., Feng X., Xiong J. (2024). Specific Lineage Transition of Tumor-Associated Macrophages Elicits Immune Evasion of Ascitic Tumor Cells in Gastric Cancer with Peritoneal Metastasis. Gastric Cancer.

[89] Mano Y., Igarashi Y., Komori K., Hashimoto I., Watanabe H., Takahashi K., Kano K., Fujikawa H., Yamada T., Himuro H. (2025). Characteristics and clinical significance of immune cells in omental milky spots of patients with gastric cancer. Front. Immunol..

[90] Fucà G., Cohen R., Lonardi S., Shitara K., Elez M.E., Fakih M., Chao J., Klempner S.J., Emmett M., Jayachandran P. (2022). Ascites and Resistance to Immune Checkpoint Inhibition in dMMR/MSI-H Metastatic Colorectal and Gastric Cancers. J. Immunother. Cancer.

[91] Berger J.M., Preusser M., Berghoff A.S., Bergen E.S. (2023). Malignant Ascites: Current Therapy Options and Treatment Prospects. Cancer Treat. Rev..

[92] Lei Y., Cai S., Zhang C.-D., Li Y.-S. (2024). The Biological Role of Extracellular Vesicles in Gastric Cancer Metastasis. Front. Cell Dev. Biol..

[93] Yamaguchi H., Miyazaki M. (2025). Cell Biology of Cancer Peritoneal Metastasis: Multiclonal Seeding and Peritoneal Tumor Microenvironment. Cancer Sci..

[94] Hu Q.-J., Ito S., Yanagihara K., Mimori K. (2018). Molecular Mechanism of Peritoneal Dissemination in Gastric Cancer. J. Cancer Metastasis Treat..

[95] Satala C.B., Kovacs Z., Bara T., Jung I., Gurzu S. (2023). Signet-Ring Cell Squamous Cell Carcinoma: A Biphenotypic Neoplasm of the Gastro-Esophageal Junction with Uncertain Biological Potential: Case Report and Literature Review. Int. J. Mol. Sci..

[96] Kim H.-I., Badgwell B.D. (2025). Peritoneal Oligometastasis in Gastric Cancer: Diagnostic Strategies, Patient Selection, and Emerging Therapeutic Approaches. J. Gastric Cancer.

[97] Saito S., Yamaguchi H., Saito A., Kaneko Y., Ohzawa H., Yokota S., Kitayama J. (2025). Advances in Intraperitoneal Chemotherapy for Gastric Cancer Patients with Peritoneal Metastases: Current Status of Treatment and Institutional Insights. J. Clin. Med..

[98] Jony J.H., Ranjbar S., Prajapati R., Eslami S.M., Zhen Z., Darji M., Zhu X., Lu X. (2025). Physiological Considerations and Delivery Strategies for Targeting Tumors through Intraperitoneal Delivery. Pharm. Res..

[99] Li W., Huang X., Han X., Zhang J., Ma B., Yin Z., Wang Y., Gao L., Shi J., Chen H. (2025). Sequential HIPEC, Claudin18.2-Targeted Therapy, and CapeOx Chemotherapy Leading to Resolution of Peritoneal Metastases and Curative Resection in Gastric Cancer: A Case Report and Literature Review. Front. Immunol..

[100] Restle D., Amador-Molina A., Misawa K., Banerjee S., Ku G., Adusumilli P.S. (2025). Intraperitoneal CAR T-Cell Therapy for Peritoneal Carcinomatosis from Gastroesophageal Cancer: Preclinical Investigations to a Phase I Clinical Trial (NCT06623396). J. Immunother. Cancer.

[101] Qian S., Villarejo-Campos P., García-Olmo D. (2021). The Role of CAR-T Cells in Peritoneal Carcinomatosis from Gastric Cancer: Rationale, Experimental Work, and Clinical Applications. J. Clin. Med..

[102] Tajik F., Eyob B., Khan A.M., Radhakrishnan V.K., Senthil M. (2025). Iterative Intraperitoneal Chemotherapy in Gastric Cancer Peritoneal Carcinomatosis. Cancers.

[103] Bai L., Guan Y., Zhang Y., Gu J., Ni B., Zhang H., Aimaiti M., Wang S., Yue B., Xia X. (2024). Effectiveness of Peritoneal Lavage Fluid Circulating Tumour Cells and Circulating Tumour DNA in the Prediction of Metachronous Peritoneal Metastasis of Gastric Cancer (pT4NxM0/pT1-3N+M0) after Radical Resection: Protocol of a Prospective Single-Centre Clinical Study. BMJ Open.

[104] Han H.S., Lee K.-W. (2024). Liquid Biopsy: An Emerging Diagnostic, Prognostic, and Predictive Tool in Gastric Cancer. J. Gastric Cancer.

[105] Kim S., Lee H.H., Song K.Y., Seo H.S. (2024). Peritoneal Washing Cytology Positivity in Gastric Cancer: Role of Lymph Node Metastasis as a Risk Factor. J. Gastric Cancer.

[106] Acs M., Piso P., Glockzin G. (2024). Peritoneal Metastatic Gastric Cancer: Local Treatment Options and Recommendations. Curr. Oncol..

[107] Roensholdt S., Detlefsen S., Mortensen M.B., Graversen M. (2023). Response Evaluation in Patients with Peritoneal Metastasis Treated with Pressurized IntraPeritoneal Aerosol Chemotherapy (PIPAC). J. Clin. Med..

[108] van Hootegem S.J.M., Guchelaar N.A.D., van der Sluis K., Triemstra L., Mönig S.P., Rawicz-Pruszyński K., Rosati R., Morgagni P., Erodotou M., Solaini L. (2025). Staging Laparoscopy for Gastric Cancer: European Consensus. Br. J. Surg..

[109] Li Z., Wong L.C.K., Sultana R., Lim H.J., Tan J.W.-S., Tan Q.X., Wong J.S.M., Chia C.S., Ong C.-A.J. (2022). A Systematic Review on Quality of Life (QoL) of Patients with Peritoneal Metastasis (PM) Who Underwent Pressurized Intraperitoneal Aerosol Chemotherapy (PIPAC). Pleura Peritoneum.

[110] Parisi A., Porzio G., Ficorella C. (2020). Multimodality Treatment in Metastatic Gastric Cancer: From Past to Next Future. Cancers.

[111] Fu C., Zhang B., Guo T., Li J. (2024). Imaging Evaluation of Peritoneal Metastasis: Current and Promising Techniques. Korean J. Radiol..

[112] Ruiz Hispán E., Pedregal M., Cristobal I., García-Foncillas J., Caramés C. (2021). Immunotherapy for Peritoneal Metastases from Gastric Cancer: Rationale, Current Practice and Ongoing Trials. J. Clin. Med..

[113] Fallah M., Detlefsen S., Ainsworth A.P., Fristrup C.W., Mortensen M.B., Pfeiffer P., Tarpgaard L.S., Graversen M. (2022). Importance of Biopsy Site Selection for Peritoneal Regression Grading Score (PRGS) in Peritoneal Metastasis Treated with Repeated Pressurized Intraperitoneal Aerosol Chemotherapy (PIPAC). Pleura Peritoneum.

[114] Zhao D., Yue P., Wang T., Wang P., Song Q., Wang J., Jiao Y. (2021). Personalized analysis of minimal residual cancer cells in peritoneal lavage fluid predicts peritoneal dissemination of gastric cancer. J. Hematol. Oncol..

[115] Lordick F., Carneiro F., Cascinu S., Fleitas T., Haustermans K., Piessen G., Vogel A., Smyth E.C., ESMO Guidelines Committee (2022). Gastric Cancer: ESMO Clinical Practice Guideline for Diagnosis, Treatment and Follow-Up. Ann. Oncol..

[116] Ajani J.A., D’Amico T.A., Bentrem D.J., Corvera C.U., Das P., Enzinger P.C., Enzler T., Gerdes H., Gibson M.K., Grierson P. (2025). Gastric Cancer, Version 2.2025, NCCN Clinical Practice Guidelines In Oncology. J. Natl. Compr. Cancer Netw..

[117] Li S., Zhang W., Yang Q., Li S., Zhou X., Wang H., Sun Y., Liu X., Ji C., Zhou J. (2026). Genomic Landscape of Paired Primary and Peritoneal Metastatic Lesions in Gastric Cancer Highlights Evolutionary Dynamics and Mutational Drivers. J. Adv. Res..

[118] Tanaka Y., Chiwaki F., Kojima S., Kawazu M., Komatsu M., Ueno T., Inoue S., Sekine S., Matsusaki K., Matsushita H. (2021). Multi-Omic Profiling of Peritoneal Metastases in Gastric Cancer Identifies Molecular Subtypes and Therapeutic Vulnerabilities. Nat. Cancer.

[119] Fleurkens-Ewals L.J.S., Tops-Welten M., Claessens C.H.B., Piek J.M.J., van Hellemond I.E.G., van der Sommen F., Lahaye M.J., de Hingh I.H.J.T., Luyer M.D.P., Nederend J. (2025). Artificial Intelligence and Radiomics Models for the Diagnosis and Prognosis of Peritoneal Metastases on Imaging: A Systematic Review and Meta-Analysis. Comput. Biol. Med..

